# Time-resolved hemodynamic responses to sentence-level speech perception, production, and self-monitoring

**DOI:** 10.1162/IMAG.a.1375

**Published:** 2026-09-21

**Authors:** Teng Ieng Leong, Aotong Li, Jian Hwee Ang, Barry Lee Reynolds, Yan Tian, Cheok Teng Leong, Chi Un Choi, Martin I. Sereno, Defeng Li, Victoria Lai Cheng Lei, Ruey-Song Huang

**Affiliations:** Centre for Cognitive and Brain Sciences, University of Macau, Taipa, Macau SAR, China; Faculty of Arts and Humanities, University of Macau, Taipa, Macau SAR, China; Faculty of Science and Technology, University of Macau, Taipa, Macau SAR, China; Faculty of Education, University of Macau, Taipa, Macau SAR, China; Department of Cognitive Science, University of California, San Diego, La Jolla, CA, United States

**Keywords:** sparse-sampling fMRI, speech perception, overt production, spatial ICA, time-resolved fMRI

## Abstract

Functional magnetic resonance imaging (fMRI) has been widely used to explore the neural mechanisms underlying speech processing. However, the intertwining of perception and production that exists in real-world scenarios remains underexplored due to challenges such as gradient noise and head motion artifacts from speaking. Previous research has often employed sparse-sampling designs, pausing image acquisition intermittently to present auditory stimuli or record overt speech. While this approach mitigates some challenges, it cannot capture continuous brain activity during speech processing and does not separate the mixed hemodynamic responses to external and self-generated speech occurring in succession. We overcame these limitations and continuously scanned 31 participants as they listened to and recited English sentences. Through independent component analysis (ICA), we decomposed each functional scan into spatially independent components (ICs), identifying task-related ICs with activations predominantly located in the superior temporal cortex, inferior frontal gyrus, and orofacial sensorimotor cortex. These ICs demonstrated time-resolved hemodynamic responses corresponding to distinct stages of speech perception, planning, and production. A linear subtraction between the IC time courses from the listening-reciting (perception-to-production) and listening (perception-only) tasks further revealed a secondary hemodynamic response to self-generated speech in the superior temporal cortex. Furthermore, we established precise temporal relationships between overt speech output and the peak, rise, and fall of hemodynamic responses for each independent component. Together, we present a methodological framework that can inform future fMRI studies on naturalistic tasks involving the perception of external auditory stimuli and monitoring of self-generated sounds.

## 1 Introduction

The ability to distinguish between sequential activations in the brain regions associated with auditory perception, production, and self-monitoring is vital for advancing our understanding of cognitive processes across various domains, particularly in language and music processing. Despite significant progress in mapping the cortical representations of language processing with functional magnetic resonance imaging (fMRI) (Price, 2012), the temporal dynamics of brain activity during naturalistic sentence-level speech processing have not been sufficiently investigated. Previous studies on sentence-level speech perception focused on the time courses of linguistic properties (Huang et al., 2013; Matchin et al., 2019), while most speech production studies investigated brain dynamics using single- or multi-word production tasks (Janssen et al., 2020; Janssen & Mendieta, 2020; Ries et al., 2013; Verwoert et al., 2025).

Investigating the overt production of sentences using fMRI presents inherent challenges. While continuous scanning has been employed in previous studies, its broader application has often been constrained by the masking effects of gradient noise on auditory inputs and head-motion artifacts (Jolly et al., 2020). To address these challenges, some researchers have used sparse-sampling fMRI designs to avoid the interference of scanner noise on speech perception and production (Blackman & Hall, 2011; Gracco et al., 2005; Perrachione & Ghosh, 2013). In a sparse-sampling fMRI experiment, image acquisition is intermittently paused to allow for auditory stimulus presentation or the recording of overt speech against a silent background (Merrett et al., 2021), which also avoids recording head-motion artifacts while speaking. However, optimized sparse-sampling fMRI designs depend on estimating precise time windows to record the maximum brain activity following stimulus presentation or speech output. Determining the exact interval between stimulus onset and the peak hemodynamic response is challenging for paradigms involving sentence processing, as the hemodynamic response begins to rise while the stimulus is still being presented.

Although the interleaved silent steady sparse-sampling method attempts to mitigate discrepancies due to varying stimulus durations (Schwarzbauer et al., 2006), it still falls short of capturing the complete temporal profile of the hemodynamic response elicited by sentences (Wise & Geranmayeh, 2016). In our recent studies, we built on these prior efforts by implementing continuous fMRI sampling with real-time auditory feedback during sentence-level speech perception and production tasks (Lei et al., 2024, 2025). These studies demonstrated that continuous sampling could resolve the phases of periodic hemodynamic fluctuations, enabling further exploration of complex spatiotemporal brain dynamics within and across regions, which are inaccessible with sparse-sampling designs. However, a persistent challenge for continuous sampling is disentangling the overlapping blood-oxygenation-level–dependent (BOLD) signals elicited by external and self-generated speech occurring in close temporal proximity. While typical experimental designs for studying the neurocognitive basis of speech processing usually consist of a brief period of either speech perception or production within each trial (Vigneau et al., 2006; Walenski et al., 2019), real-world communication often involves overlapping or sequential intervals of speech perception and production. For example, an interpreter actively listens to a spoken sentence in a source language and renders it into a target language, either simultaneously or consecutively (Hervais-Adelman et al., 2015; Paradis, 1994). In a similar vein, collaborative singing serves as a prime example of music processing that involves both external auditory input and self-monitoring, where vocalists listen to other vocalists or instrumental accompaniment while simultaneously monitoring their own vocal output to achieve harmony and synchronize their performance. Both scenarios highlight the auditory system’s capacity to process both external stimuli and self-generated sounds, which leads to temporally mixed hemodynamic responses in the auditory regions.

In this study, subjects were scanned continuously while they engaged in two tasks: listening to spoken sentences and listening to, memorizing, and reciting spoken sentences. Using independent component analysis (ICA) (Beckmann & Smith, 2004; Davis et al., 2014; Janssen & Mendieta, 2020; McKeown et al., 1998; Plante et al., 2017), we aim to decompose and identify independent brain processes associated with speech perception, planning, production, and self-monitoring. By addressing the challenges of overlapping BOLD signals, this research seeks to capture and unravel the full temporal profiles of hemodynamic responses during the speech perception-to-production process, contributing to a deeper understanding of auditory processing in real-world communication.

## 2 Methods

### 2.1 Participants

The study analyzed fMRI datasets of 31 subjects (22 females, 9 males; mean age: 23.3 ± 4 years) recruited by the Brain and Language Project at the University of Macau (Lei et al., 2024). All subjects were native Chinese (Mandarin) speakers who acquired English as a second language between the ages of 4 and 13 (mean age of acquisition = 7.1 ± 2.1 years). English proficiency was assessed based on their most recent standardized test results (an IELTS score of 6.5 or above, or an equivalent qualification). All subjects gave written informed consent in accordance with the protocols approved by the Research Ethics Committee of the University of Macau. All subjects had normal or corrected-to-normal vision and reported no history of neurological disorders.

### 2.2 Experimental procedures and setup

**Fig. 1.**
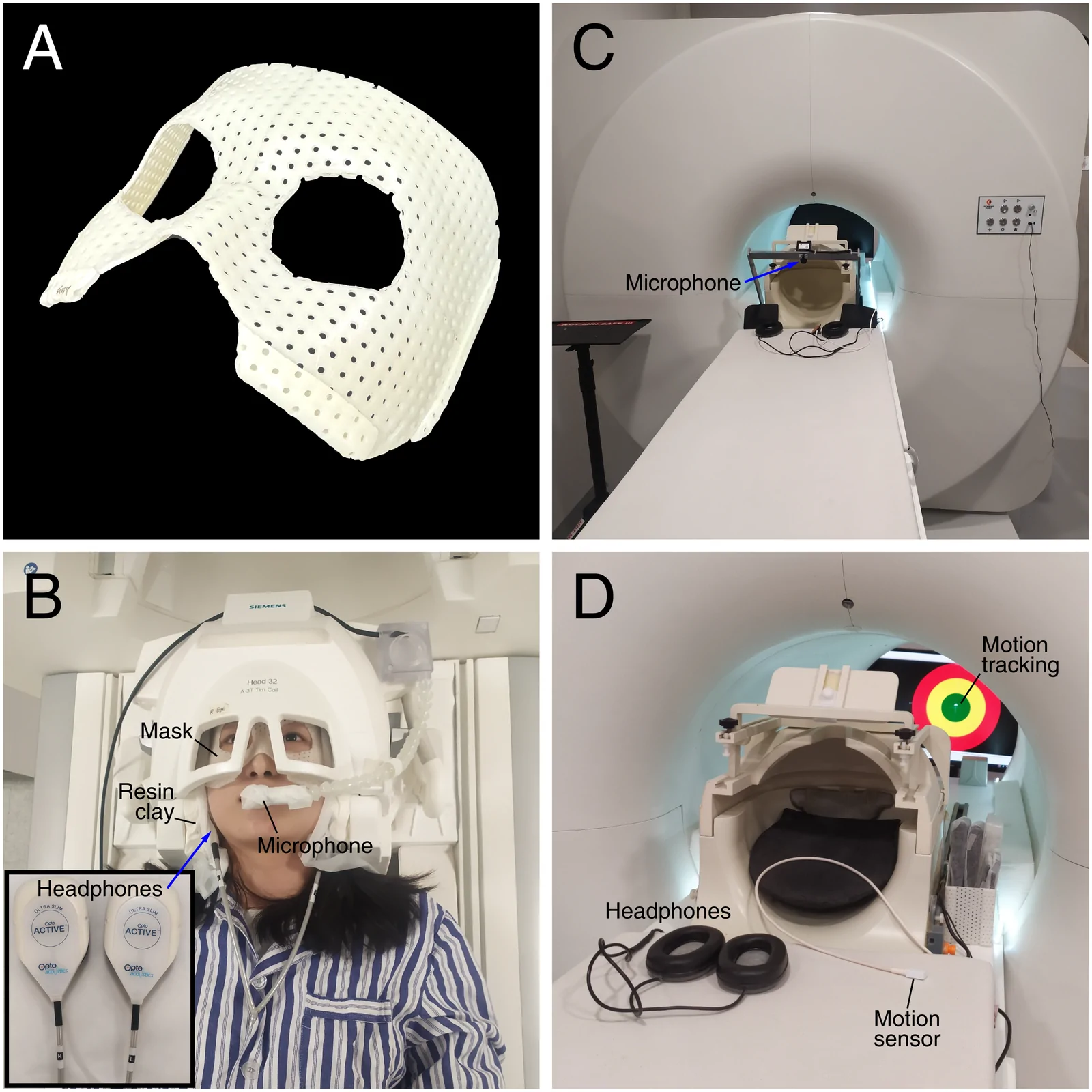
Experimental setup for speech perception and production tasks. (A) A custom-molded mask with “The phantom of the opera” style for head immobilization. (B) Demonstration of subject positioning in a Siemens 32-channel head coil. The subject wears a mask and a pair of MR-compatible headphones, with a microphone placed in front of her mouth. The gaps surrounding the mask and headphones are filled with resin clay and silicone gel. (C) An MRI simulator (bore diameter = 60 cm) and a mock coil equipped with a microphone and headphones. (D) A close-up view of the mock coil. The motion sensor is affixed to the subject’s forehead during simulator training.

To minimize head motion during overt speech production, we made an individualized facial mask for each subject using thermoplastic sheets (1.6 mm H-board, Sun Medical Products Co., Ltd.) (Fig. 1A, B). All subjects then wore their masks and completed a training session to practice speaking while maintaining head stillness in a mock scanner (Shenzhen Sinorad Medical Electronics Co., Ltd.) (Fig. 1C). During the training, real-time head motion was monitored using a sensor attached to their foreheads (MoTrak, Psychology Software Tools, Inc.) (Fig. 1D). Subjects would hear a warning sound (“ding”) if their head motion exceeded 1 mm in translation or 1° in rotation, allowing them to monitor their head position while speaking.

Prior to the fMRI session, subjects would wear a mask and earplugs, with their ears fully covered by a set of MR-compatible headphones (OptoACTIVE II, OptoAcoustics Ltd.), and then lay supine with their heads supported by resin clay in a head coil (Fig. 1B). The mask and headphones were further stabilized by filling resin clay and silicone gel in the gap between the head and the inner wall of the head coil, which also effectively attenuated scanner noise.

To ensure clear and intelligible delivery of auditory stimuli during fMRI experiments, a dedicated noise reduction system was implemented. The MR-compatible headphones and microphone (FORMRI-III, OptoAcoustics Ltd.) were connected to a real-time active noise cancellation system via fiber optic cables passing through a waveguide connecting the scanner room and control room. This system analyzed the power spectrum of gradient noise from an echo planar imaging (EPI) pulse sequence and actively cancelled the noise, effectively suppressing scanner-generated interference during speech perception and production tasks.

In addition to active noise cancellation, passive attenuation was achieved through the combined use of earplugs, resin clay, foam padding, and an acoustic foam (4.5 cm thick) lining the scanner bore. These measures substantially reduced ambient gradient noise. All subjects reported no difficulty in hearing or understanding the auditory stimuli, including external and self-generated speech, throughout each functional scan. Together, these integrated measures for countering head motion and scanner noise enabled continuous functional imaging during sentence-level speech perception and overt reciting tasks.

### 2.3 Experimental design

All subjects participated in 12 scans of six sentence-level perception and production tasks for both Chinese and English sessions (Lei et al., 2024). Of the 12 scans comprising six language tasks, the present study focused on the listening and listening-reciting tasks from the English session. Each task was performed in two 256-s functional scans with a rapid phase-encoded fMRI design (also known as the periodic event-related design; Chen et al., 2019; Lei et al., 2024), each consisting of sixteen 16-s consecutive trials, that is, 16 cycles/scan. Within each trial of the listening task, subjects passively listened to a spoken sentence for 5 s (perception phase: 0–5 s), followed by 11 s of rest (5–16 s). Within each trial of the listening-reciting task, subjects passively listened to and memorized a spoken sentence for 5 s (perception phase: 0–5 s), followed by 5 s of reciting from memory (production phase: 5–10 s), and 6 s of rest (10–16 s). Visual cues, including an ear icon for the perception phase and a mouth icon for the production phase, were presented at the center of a rear-view LCD screen. All stimuli were systematically constructed with 15–17 syllables per sentence, following the subject-verb-object structure, for example, “She has posted photographs of her friends on bulletin boards.” All stimuli were built from high-frequency vocabulary sourced from the *Longman Communication 3000 list* (https://www.lextutor.ca/freq/lists_download/longman_3000_list.pdf) and the *Collins English Dictionary* (https://www.collinsdictionary.com/dictionary/english), and validated for linguistic appropriateness by a professor of linguistics.

### 2.4 Image acquisition

All images were acquired with a 32-channel head coil in a 3T MRI scanner (Siemens MAGNETOM Prisma) at the Centre for Cognitive and Brain Sciences, University of Macau. For functional imaging, whole-brain functional images were acquired using a blipped-CAIPIRINHA simultaneous multi-slice (SMS), single-shot EPI sequence with the following parameters: acceleration factor = 5; interleaved ascending slice order; repetition time (TR) = 1000 ms; echo time (TE) = 30 ms; flip angle = 60°; 55 axial slices; field of view (FOV) = 192 × 192 mm²; matrix size = 64 × 64; voxel size = 3 × 3 × 3 mm³; bandwidth = 2368 Hz/Px. Each functional scan comprised 256 volumes (excluding 6 dummy volumes), resulting in an effective scan time of 256 s per scan. Additionally, two scans of T1-weighted structural images were acquired, matching the slice center and orientation of functional scans, using an MPRAGE sequence (TR = 2300 ms; TE = 2.26 ms; inversion time (TI) = 900 ms; flip angle = 8°; 256 axial slices; FOV = 256 × 256 mm²; matrix = 256 × 256; voxel size = 1 × 1 × 1 mm³; bandwidth = 200 Hz/Px; scan time = 234 s).

### 2.5 Image preprocessing

**Fig. 2.**
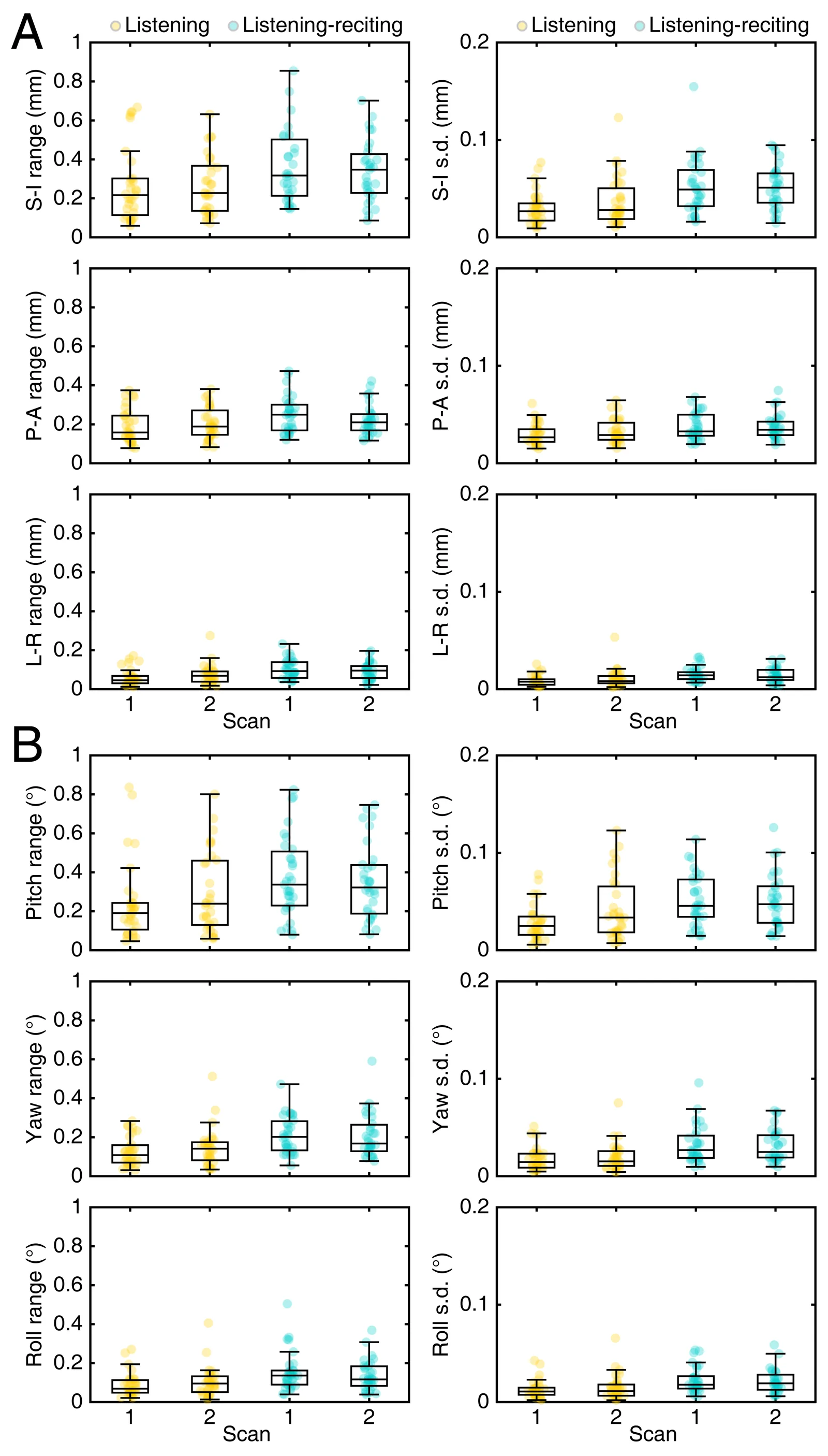
Distribution of motion parameters in six degrees of freedom. (A) Head displacement in the superior-inferior (S-I), posterior-anterior (P-A), and left-right (L-R) directions. (B) Head rotation in pitch, yaw, and roll. Left column: distribution of the range (maximum - minimum) of motion within each scan across subjects. Right column: distribution of the standard deviation (s.d.) of motion parameters within each scan across subjects. Each color marker indicates the data point from a single subject (*N* = 31).

All raw images (DICOM .ima files) were converted to Analysis of Functional NeuroImages (AFNI; https://afni.nimh.nih.gov) .BRIK files using the AFNI *to3d* command. Functional images were motion-corrected by registering the volumes in all scans to the first volume of the seventh functional scan (immediately after a structural scan) using the AFNI *3dvolreg* command, yielding motion parameters in six degrees of freedom for each scan (Fig. 2). Subsequently, the motion-corrected functional images were coregistered to the structural images using the *csurf* package (https://pages.ucsd.edu/~msereno/csurf/) (Sereno et al., 2022). For each subject, two sets of T1-weighted structural images were aligned and averaged prior to cortical surface reconstruction using FreeSurfer 7.2 (https://surfer.nmr.mgh.harvard.edu) (Dale et al., 1999; Fischl, Sereno, & Dale, 1999).

### 2.6 Audio recording and analysis

The soundtracks of auditory stimuli, speech output, and Transistor-Transistor Logic (TTL) signals in sync with the EPI pulse sequence were recorded simultaneously using the OptiMRI 3.1 software (OptoAcoustics Ltd.). The timings of the onset and offset of speech production in the listening-reciting task were manually identified from the speech-output channel using the Audacity software (https://www.audacityteam.org).

### 2.7 Independent component analysis

**Fig. 3.**
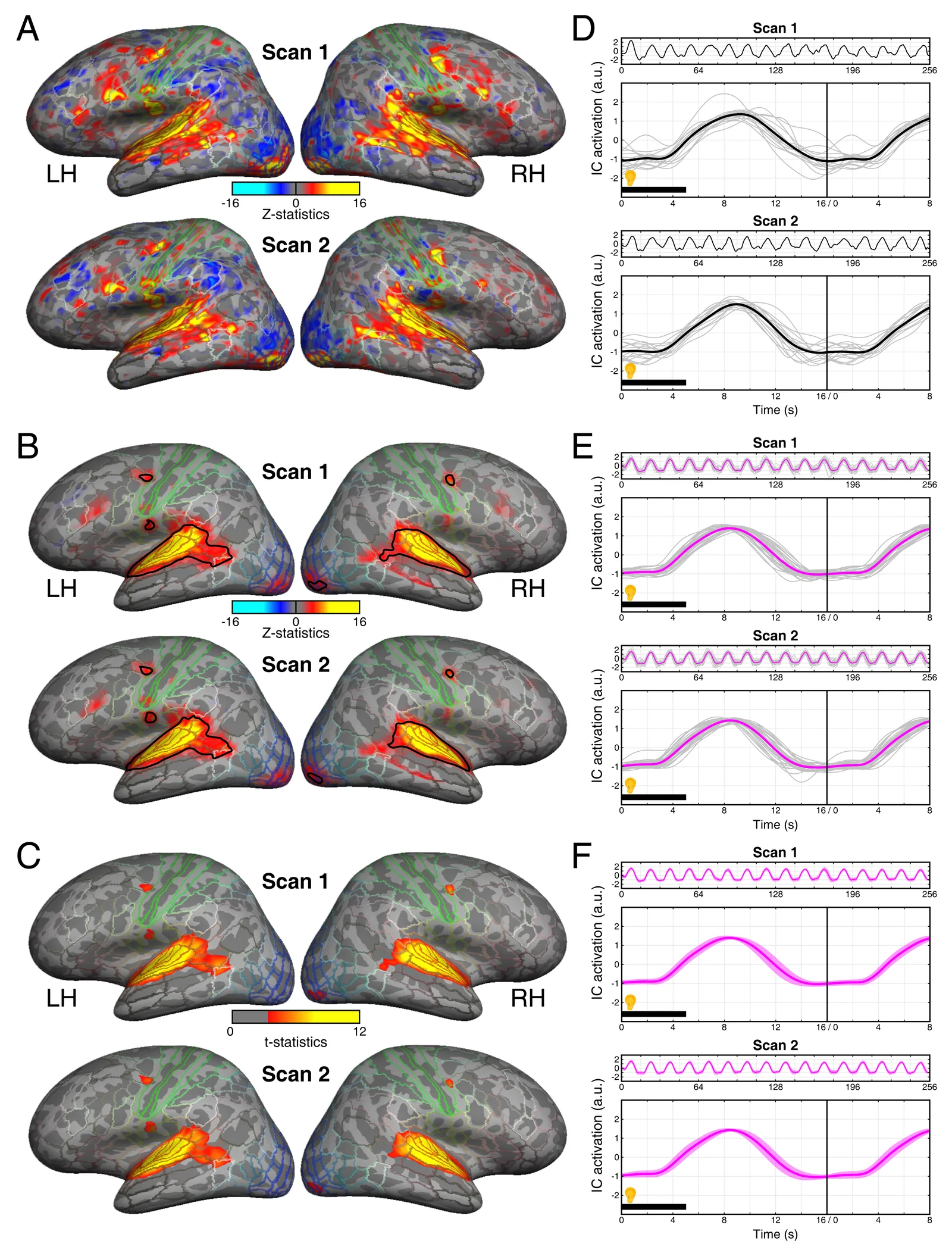
Spatial maps and time courses of the STC IC in the listening task. (A) Spatial maps of the STC IC derived from two scans of Subject 1. Z-statistics threshold = 1. LH: left hemisphere; RH: right hemisphere. The maps are overlaid with the borders of regions of interest (ROIs) defined in the HCP-MMP1 atlas (Glasser et al., 2016). (B) Group-average Z-statistics maps (N = 31) of the STC IC derived from two scans. Z-statistics threshold = 1. Black contours indicate the mean Z-statistics are significantly higher than one, as determined by one-sample t-tests (t(30) > 3.385, p < 0.001, one-tailed; p < 0.01, cluster corrected) in (C). (D) Time courses associated with Subject 1’s IC maps in (A). a.u.: arbitrary unit. The first and third panels show the 256-s time courses for both scans from Subject 1. The second and fourth panels show the time courses in 24-s epochs (gray curves) and their average (black curve) within each scan. (E) Single-subject and group-average time courses. The first and third panels show the 256-s time courses from 31 subjects (gray curves) and their average (magenta curves) for both scans. The second and fourth panels show the time courses in 24-s epochs averaged within each subject (gray curves) and across 31 subjects (magenta curves) for both scans. (F) Mean ± 1 standard deviation (magenta curve and shading) of time courses from each corresponding panel in (E).

**Fig. 4.**
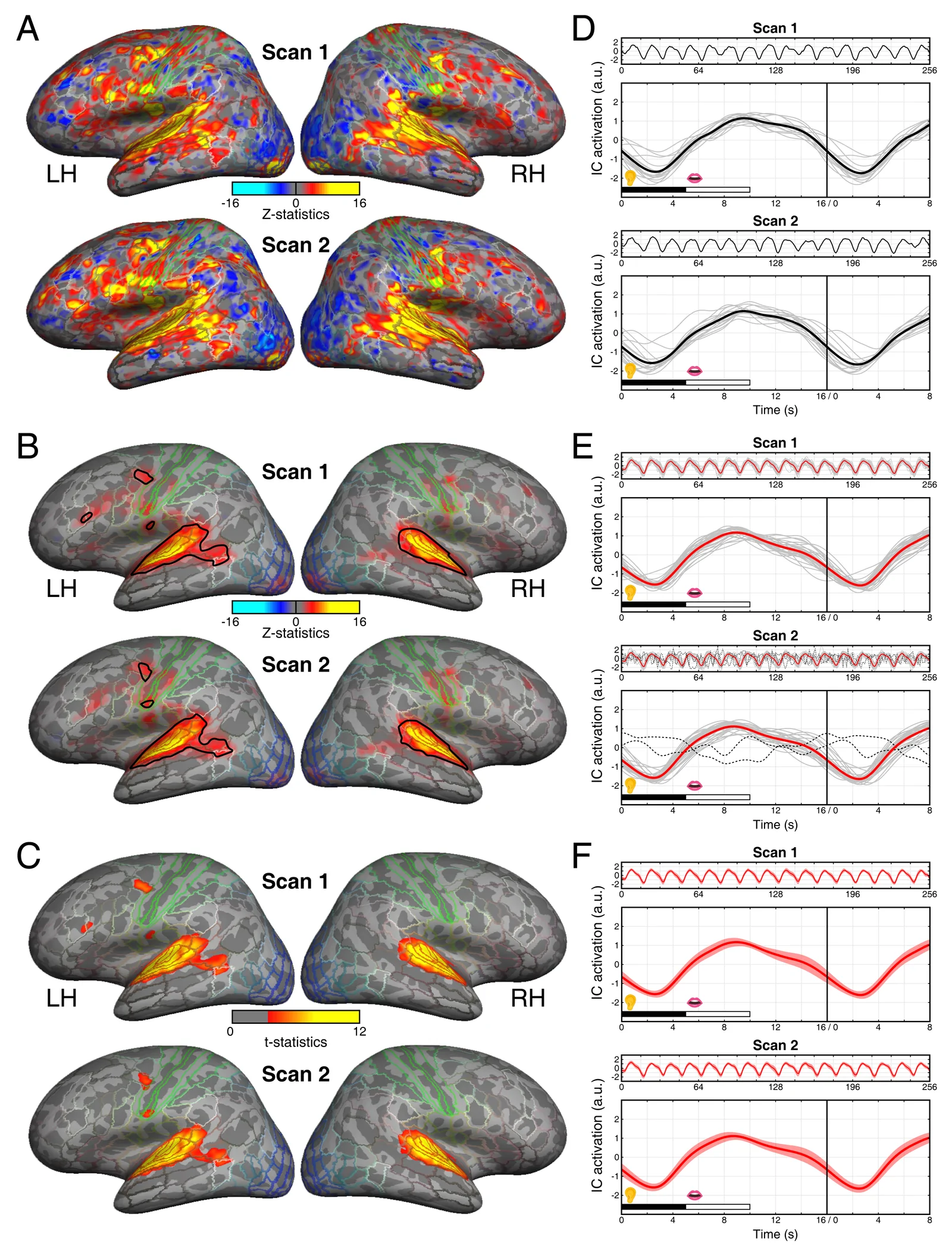
Spatial maps and time courses of the STC IC in the listening-reciting task. (A) Spatial maps of the STC IC from two scans of Subject 1. (B) Group-average Z-statistics maps (N = 31) of the STC IC derived from two scans. (C) One-sample t-tests (p < 0.01, cluster corrected) of the group-average maps in (B). (D) Time courses associated with Subject 1’s IC maps in (A). (E) Single-subject (gray) and group-average (red) time courses. First and third panels: 256-s time courses of 31 subjects. Second and fourth panels: 24-s time courses of 31 subjects. Dotted black curves: two outliers excluded from the group averaging of time courses (N = 29 for Scan 2). (F) Mean ± 1 standard deviation (red curve and shading) of time courses from each corresponding panel in (E). Other conventions follow Figure 3.

**Fig. 5.**
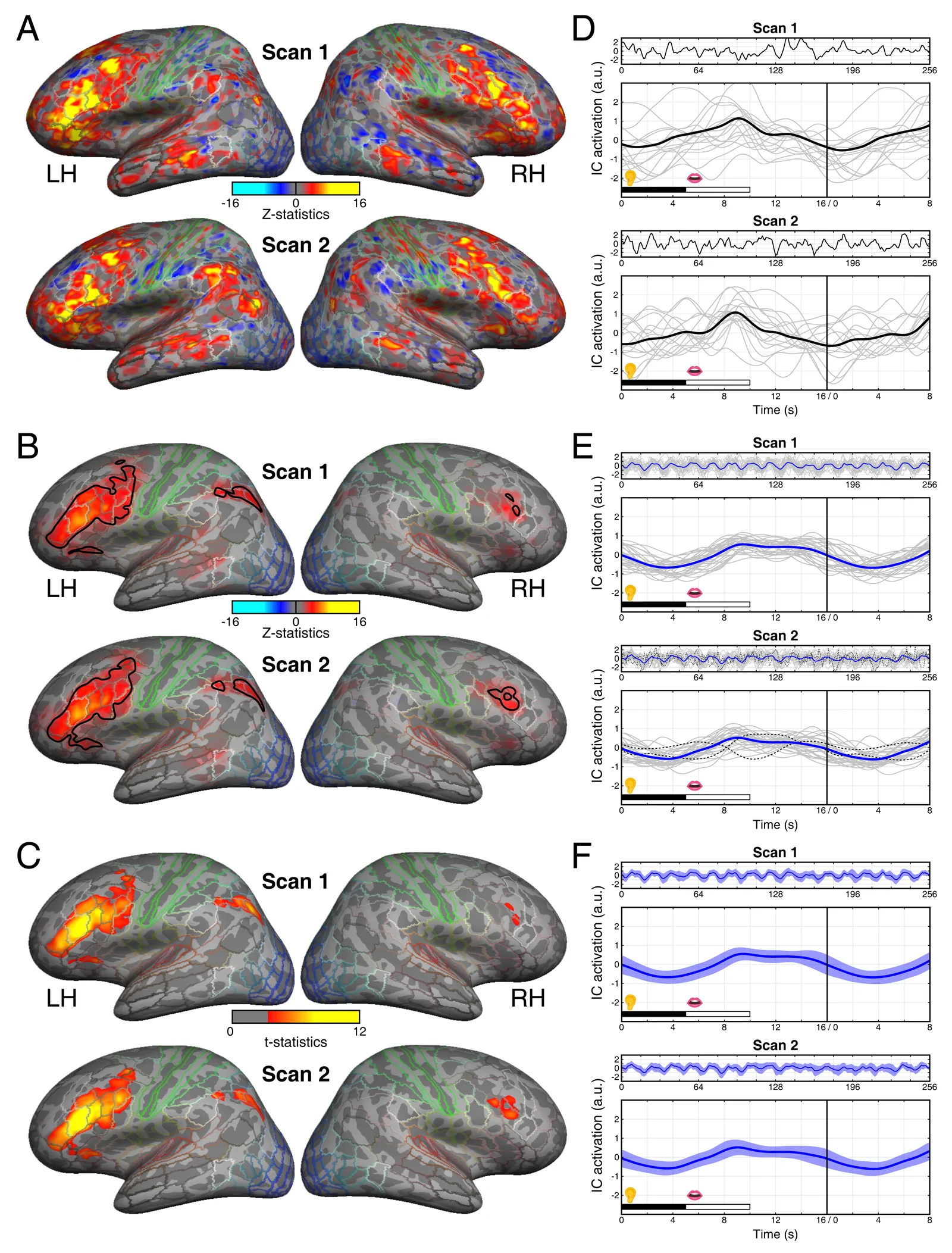
Spatial maps and time courses of the IFG IC in the listening-reciting task. (A) Spatial maps of the IFG IC from two scans of Subject 1. (B) Group-average Z-statistics maps (N = 31) of the IFG IC derived from two scans. (C) One-sample t-tests (p < 0.01, cluster corrected) of the group-average maps in (B). (D) Time courses associated with Subject 1’s IC maps in (A). (E) Single-subject (gray) and group-average (blue) time courses. First and third panels: 256-s time courses of 31 subjects. Second and fourth panels: 24-s time courses of 31 subjects. Dotted black curves: two outliers excluded from the group averaging of time courses (N = 29 for Scan 2). (F) Mean ± 1 standard deviation (blue curve and shading) of time courses from each corresponding panel in (E). Other conventions follow Figure 3.

**Fig. 6.**
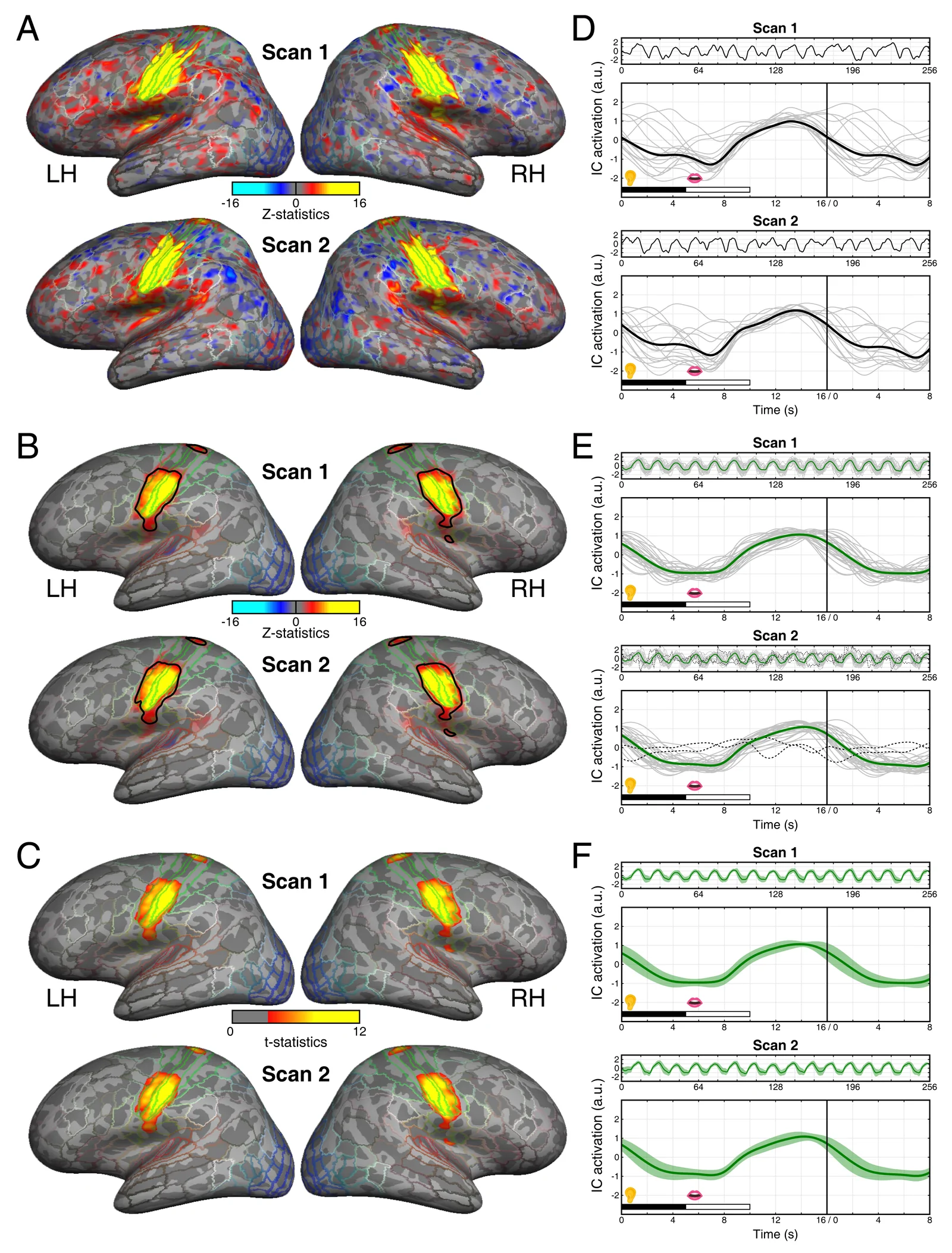
Spatial maps and time courses of the SMC IC in the listening-reciting task. (A) Spatial maps of the SMC IC from two scans of Subject 1. (B) Group-average Z-statistics maps (N = 31) of the SMC IC derived from two scans. (C) One-sample t-tests (p < 0.01, cluster corrected) of the group-average maps in (B). (D) Time courses associated with Subject 1’s IC maps in (A). (E) Single-subject (gray) and group-average (green) time courses. First and third panels: 256-s time courses of 31 subjects. Second and fourth panels: 24-s time courses of 31 subjects. Dotted black curves: two outliers excluded from the group averaging of time courses (N = 29 for Scan 2). (F) Mean ± 1 standard deviation (green curve and shading) of time courses from each corresponding panel in (E). Other conventions follow Figure 3.

Following motion correction and registration, the complete 4D dataset (64 × 64 × 55 × 256 data points) of each functional scan from individual subjects was subjected to probabilistic independent component analysis (Beckmann & Smith, 2004) using the FSL MELODIC toolbox (Version 3.15; https://fsl.fmrib.ox.ac.uk/fsl/docs/resting_state/melodic.html). The analysis resulted in a spatial map (64 × 64 × 55 points of Z-statistics) and a time course (256 points) for each independent component (IC) decomposed from each functional scan of each subject. Each IC spatial map (voxel-based) was displayed on the cortical surface of each subject using the FreeSurfer *paint* command, yielding a paint file (.w) consisting of a Z-statistics value at each vertex on the cortical surface (Figs. 3–6, A). This subject-level surface-based IC spatial map (“IC map”) was morphed and resampled to the cortical surface of the template brain *fsaverage* (Supplementary Figs. S1–S8) using the FreeSurfer *mri_surf2surf* command (https://freesurfer.net/fswiki/mri_surf2surf).

### 2.8 Time courses of independent components

**Fig. 7.**
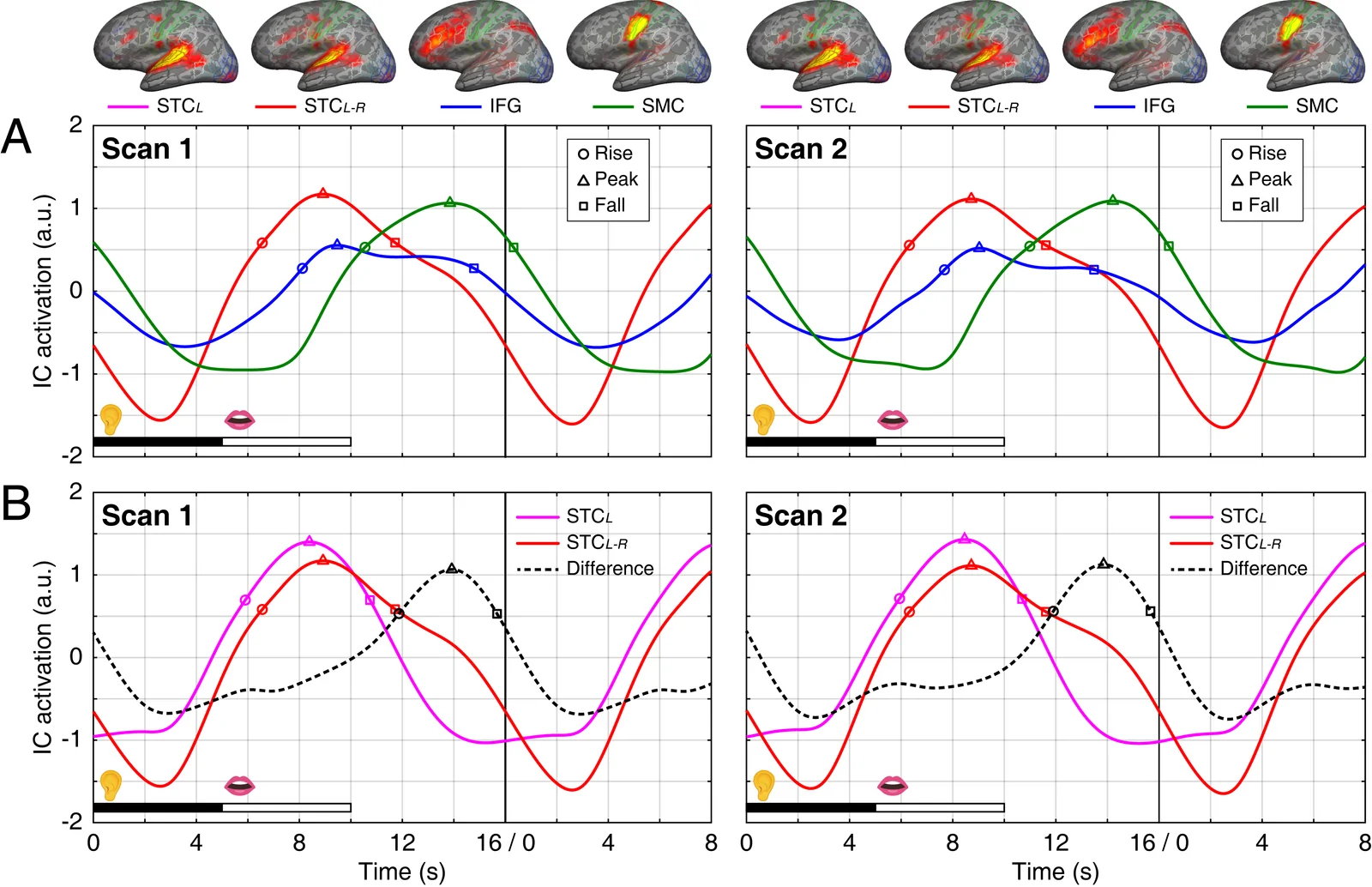
Group-average IC activation time courses. (A) Overlay of group-average time courses of the ICs identified for the listening-reciting task (redrawn from Figs. 4–6). (B) Each black dashed curve indicates the difference between the time courses of the STC*_L-R_* IC (listening-reciting task, red curve; redrawn from Fig. 4) and STC*_L_* IC (listening task, magenta curve; redrawn from Fig. 3). The top row shows the group-average IC spatial maps in the left hemisphere (redisplayed from Figs. 3–6) for Scan 1 and Scan 2, respectively. The color coding for group-average IC time courses is consistent with that in Figures 3–6. The circle, triangle, and square indicate the timings of the rise, peak, and fall in the group-average hemodynamic response profile. Exact values are provided in Table 1.

**Fig. 8.**
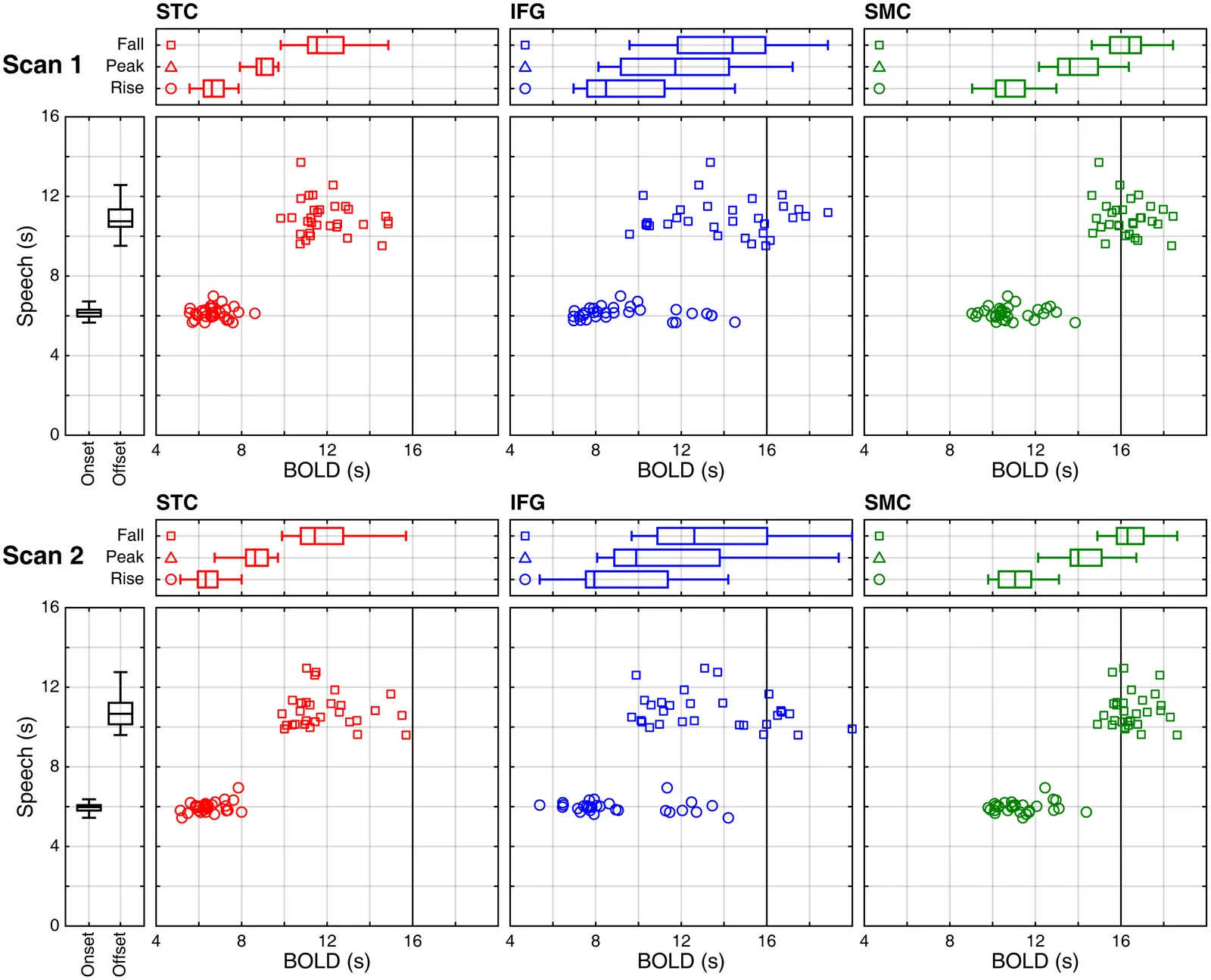
Distributions of key timings of speech output and hemodynamic responses in the listening-reciting task. For each scan, the topmost horizontal panels display the distributions of the rise, peak, and fall times of the hemodynamic responses (BOLD) in the STC (red), IFG (blue), and SMC (green) ICs. The leftmost vertical panels show the distributions of speech onset and offset times. *N* = 31 for Scan 1 and *N* = 29 for Scan 2. In the scatter plots, each marker indicates a subject’s data point. Open circles: joint distribution of the timings of speech onset and hemodynamic response rise across subjects. Open squares: joint distribution of the timings of speech offset and hemodynamic response fall across subjects.

For each subject *S*, an IC time course *X*(*t*), *t* ∈ {1, 2,…, 256} s, was upsampled 100 times to *X’*(*t’*), *t’* ∈ {0, 0.01, 0.02,…, 256} s, using the cubic spline interpolation algorithm implemented in the MATLAB *interp1* function. Note that the data point at *t* = 0 was duplicated from that at *t* = 1 prior to the interpolation. The upsampled time course was then segmented into 16 overlapping 24-s epochs, *${X^{\prime }}_{E}\left({t^{\prime }}_{e}\right)$*, *E* ∈ {1, 2,…, 16} and ${t^{\prime }}_{e}\in \left\{0,\;0.01,\;0.02,\;\ldots ,\;24\right\}\;s$ . Each epoch began at the onset (0 s) of a 16-s trial and continued for 8 s into the subsequent trial, ensuring that the complete hemodynamic response profile was included (Figs. 3–6, D–F). The data points in the 16 to 24-s range of the last (16th) epoch were obtained by averaging the corresponding data points from the first 15 epochs. For each IC time course, 16 epochs ${X^{\prime }}_{E}\left({t^{\prime }}_{e}\right)$ were averaged within each subject, resulting in ${X^{\prime }}_{S}\left({t^{\prime }}_{e}\right)$. All trials from each scan were taken into account for the average, and no trials were excluded. A group-average epoch ${X^{\prime }}_{G}\left({t^{\prime }}_{e}\right)$ was obtained by further averaging epochs ${X^{\prime }}_{S}\left({t^{\prime }}_{e}\right)$, *S* ∈ {1, 2,…, 31}, across subjects (Fig. 7). For each IC time course, the timings of the peak (maximum point at ${t^{\prime }}_{p}$, measured from the zero baseline), rise (ascending half-maximum point at ${t^{\prime }}_{r}$), and fall (descending half-maximum point at ${t^{\prime }}_{f}$), as well as the activation duration (full width at half maximum; FWHM = ${t^{\prime }}_{f}-{t^{\prime }}_{r}$), were identified from the hemodynamic response in each within-subject-average epoch, ${X^{\prime }}_{S}\left({t^{\prime }}_{e}\right)$, and each group-average epoch, ${X^{\prime }}_{G}\left({t^{\prime }}_{e}\right)$ (Figs. 7, 8).

### 2.9 Selection of independent components

We performed a subjective selection of individual ICs based on the consistency of activation patterns in IC maps and the presence of periodic activations in each IC. All ICs first went through initial labeling via visual inspection of the generated IC maps from each scan in each subject (Liu et al., 2019; McKeown et al., 1998). This approach is commonly referred to as manual annotation in supervised learning. Each IC map was then overlaid with the borders of regions of interest (ROIs) defined in the HCP-MMP1 atlas for precise localization and interpretation of activations (Glasser et al., 2016).

Drawing on sequentially activated regions in our and other studies (Castellucci et al., 2022; Lei et al., 2024), we hypothesized that we would find one primary IC for speech perception for the listening task, as well as one primary IC for each stage of speech perception, planning, and production for the listening-reciting task. For each selected IC cluster, we computed a vertex-wise average of Z-statistics across all single-subject IC maps displayed on the cortical surface of the standard subject *fsaverage*. The resulting surface-based group-average IC maps revealed consistent activations that were spatially aligned across subjects (Fischl et al., 1999; Sereno et al., 2022; Sood & Sereno, 2016). Spatial misalignment or a mix of random positive and negative Z-statistics at a location would result in weak to absent activations in the group-average IC map. To assess the spatial reliability of activation patterns, we used a one-sample t-test to determine if the mean of Z-statistics was significantly greater than one (standard deviation) at each vertex in the group-average IC map. Statistically significant regions were delineated with black borders on the cortical surface (t(30) > 3.385, p < 0.001, one-tailed; multiple comparisons via surface-based cluster exclusion, p < 0.01, corrected).

The group-average IC map serves as a baseline model (i.e., reference map) for post-hoc IC validation through correlation analysis. For each IC cluster, we computed Pearson’s correlation coefficients to assess the spatial similarity between: (1) IC maps from the first and second scans of each subject; and (2) each single-subject IC map and the group-average IC map. A correlation coefficient was computed based on the Z-statistics values across 163842 vertices per hemisphere for each pair of maps under comparison (Supplementary Tables S1–S5).

Once the spatial reliability and similarity of IC maps were tested, we verified whether the ICs in each cluster exhibited consistent time courses across subjects. As the current study used a periodic event-related design, we visually inspected IC time courses and power spectra for periodic signals at a task frequency of 16 cycles/scan. For each IC cluster, group-average time courses were used as reference waveforms to detect irregular time courses from individual subjects.

Beyond the three IC clusters, other ICs were not further analyzed in the current study because: (1) their spatial activation patterns were not consistently identified in all subjects; (2) their time courses did not exhibit persistent periodic activations at the task frequency.

## 3 Results

### 3.1 Head motion analysis

Our comprehensive procedures and meticulous setup for fMRI experiments (Fig. 1) successfully constrained the range (maximum - minimum) of head motion to less than 1 mm in translation and 1° in rotation within each scan across tasks and subjects (Fig. 2). The efficacy of simulator training and head-constraining measures was particularly evident in the displacements observed in the left-right (median range < 0.1 mm) and posterior-anterior (median range < 0.25 mm) directions, as well as the minimal rotation in roll and yaw (median range < 0.2°). However, minor head motions were observed in the superior-inferior direction (median range < 0.4 mm) and in pitch (median range < 0.4°), which were slightly more pronounced during the listening-reciting task. The high consistency of these motion mitigation strategies across subjects was confirmed by small variations (standard deviations) in motion parameters, which were less than 0.1 mm in translation and less than 0.1° in rotation in most scans (Fig. 2).

### 3.2 Independent brain processes

#### 3.2.1 Speech perception (listening task)

Across 31 subjects, independent component analysis on each functional scan of the listening task resulted in 78.2 ± 8.7 and 82 ± 9.3 ICs for Scan 1 and Scan 2, respectively. In Subject 1, an IC exhibiting speech perception-related activations predominantly located in the superior temporal cortex (STC) was identified from both scans (Fig. 3A). The STC IC map shows activations spanning the posterior lateral sulcus, superior temporal gyrus (STG), and the middle to posterior portions of the superior temporal sulcus (mid-post STS) in both hemispheres. Furthermore, smaller and weaker activation clusters were found in the premotor cortex (PMC), inferior frontal gyrus (IFG), and parietal operculum. These activation patterns were highly consistent across Scan 1 and Scan 2 in individual subjects (see IC maps of Subjects 2–31 in Supplementary Figs. S1 and S2), as determined by vertex-wise Pearson’s correlation coefficients (Supplementary Table S1).

Surface-based group averaging across individual IC maps revealed regions that were consistently activated across subjects (Fig. 3B), as evidenced by a high correlation between each single-subject map and the group-average map of the STC IC cluster (Supplementary Table S2). The black contours in Figure 3B outline statistically significant regions as assessed by a vertex-wise one-sample t-test to a mean Z-statistics value of 1 (t(30) > 3.385, p < 0.001, one-tailed; multiple comparisons via surface-based cluster exclusion, p < 0.01, corrected; Fig. 3C). The results showed a large contiguous cluster extending from the posterior lateral sulcus to mid-post STS in each hemisphere, including areas RI, PSL, STV, TPOJ1, PHT, STSdp, STSda, TA2, PI, 52, MBelt, A1, LBelt, PBelt, A4, and A5 according to the HCP-MMP1 atlas (Glasser et al., 2016). Additionally, smaller activation clusters were found in area 55b in the premotor cortex and area OP4 in the parietal operculum.

The STC IC time courses consistently exhibited periodic BOLD signals (16 cycles/scan) in response to external speech in both 256-s scans within and across subjects (first and third panels in Fig. 3D, E, F). Within the 16-s trial cycle, the average time courses displayed a roughly bell-shaped temporal profile (second and fourth panels in Fig. 3D, E, F).

#### 3.2.2 Speech perception and production (listening-reciting task)

Across 31 subjects, independent component analysis on each functional scan of the listening-reciting task resulted in 101.5 ± 18.7 and 103 ± 17.9 ICs for Scan 1 and Scan 2, respectively. Three primary IC clusters associated with the speech perception, planning, and production processes were identified in both scans within and across subjects (Figs. 4–6). The first IC (STC) cluster exhibited activation patterns similar to those of the STC IC identified for the listening task, with additional small clusters of activations identified in frontal areas IFSa and IFSp (Fig. 4A, B, C). The second IC cluster exhibited statistically significant activations predominantly located in the inferior frontal gyrus (IFG) of the left hemisphere, including areas p47r, IFSa, IFSp, IFJa, 44, 6r, IFJp, PEF, 55b, 8Av, 8C, p9-46v, 47I, AVI, and FOP5 (Fig. 5A, B, C). Another smaller cluster of significant activation was found in the posterior parietal cortex, including areas IP1, IP2, and LIPd. The third IC cluster exhibited statistically significant activations predominantly located in the orofacial and respiratory (trunk) representations of the sensorimotor cortex (SMC, areas 4, 3a, 3b, and 1; Huang et al., 2012; Huang & Sereno, 2018; Sereno & Huang, 2006), as well as a slightly weaker activation in area OP4 in the parietal operculum (Fig. 6A, B, C). Area OP4 overlaps with an auditory area, 43aud, and the secondary somatosensory cortex (S-II) defined in the CsurfMap1 atlas (Sereno et al., 2022).

Most individual IC maps within each cluster exhibit a high degree of similarity to the group-average IC map, as assessed by Pearson’s correlation coefficients (Supplementary Figs. S3–S8; Supplementary Tables S3–S5). However, several IC maps in the IFG and SMC clusters showed very low correlation (r < 0.2). The STC IC time courses exhibited periodic BOLD signals (16 cycles/scan) in response to external and self-generated speech in both scans within and across subjects (Fig. 4D, E, F), with the exception of two subjects in Scan 2. They did not exhibit clear periodic signals at the task frequency in 256-s time courses, and they were distinctly different from the group average waveforms in 24-s epochs (black dotted curves in Fig. 4E). Consequently, the IC time courses from these two subjects in Scan 2 were excluded from group averaging and subsequent timing analyses for all three IC clusters (Figs. 4–6).

Compared with STC, the IFG IC time courses exhibited delayed activations with lower amplitudes, reflecting the planning process for speech production. Finally, the SMC IC time courses exhibited the latest activations which were induced during the reciting phase. The relative timings of activations among these IC clusters are examined in the following section.

### 3.3 Time-resolved hemodynamic responses

Despite substantial overlap in time, the group-average hemodynamic response profiles of the STC, IFG, and SMC IC clusters exhibited temporal offsets that reflected the sequential brain processes during the listening-reciting task (Fig. 7A). The timings of the rise, peak, and fall of the average hemodynamic responses (Fig. 7) are summarized for each IC cluster in Table 1. The means and standard deviations of the timings of the rise, peak and fall in single-subject IC time courses are summarized in Table 2. While spatial ICA revealed different ICs associated the with speech perception, planning, and production processes, we did not identify separate ICs for processing external speech and self-generated speech in the listening-reciting task. Instead, we identified a single IC (STC*_L-R_*) with a spatial map similar to the STC*_L_* IC identified for the listening task. Compared with the STC*_L_* IC, the STC*_L-R_* IC exhibited a widened and delayed activation profile. According to the superposition principle of linear systems (Boynton et al., 2012), we assumed that the widened profile of the STC*_L-R_* IC resulted from a linear combination of hemodynamic responses to external speech and self-generated speech. The time course of the STC*_L_* IC, serving as a reference for its response to external speech, was then subtracted from the time course of the STC*_L-R_* IC (Fig. 7B). The estimated hemodynamic response (black dashed curves in Fig. 7B) peaked at 13.92 s for Scan 1 and 13.85 s for Scan 2, which closely aligned with the hemodynamic peak of the SMC IC (13.85 s for Scan 1 and 14.21 s for Scan 2; Table 1, Fig. 7A).

**Table 1.** Key timings of hemodynamic responses in the group-average IC time courses in Figure 7.

	Task	Listening	Listening-reciting			Self-monitoring
	IC	STC*_L_*	STC*_L-R_*	IFG	SMC	STC*_L-R_* – STC*_L_*
Scan 1	Rise	5.90	6.56	8.12	10.55	11.87
	Peak	8.39	8.92	9.47	13.85	13.92
	Fall	10.74	11.72	14.78	16.32	15.68
Scan 2	Rise	5.94	6.32	7.68	10.99	11.90
	Peak	8.46	8.72	9.03	14.21	13.85
	Fall	10.69	11.61	13.48	16.38	15.67

All values were measured from the trial onset and are expressed in seconds.

**Table 2.** Means and standard deviations (s.d.) of key timings of hemodynamic responses in single-subject IC time courses.

	Task	Listening	Listening-reciting		
	IC	STC*_L_*	STC*_L-R_*	IFG	SMC
Scan 1	Rise	5.95 (0.53)	6.68 (0.71)	9.38 (2.25)	10.83 (1.16)
	Peak	8.46 (0.61)	9.21 (1.2)	11.64 (2.68)	14.06 (1.11)
	Fall	10.79 (0.67)	12 (1.37)	14.01 (2.62)	16.34 (1.06)
Scan 2	Rise	5.94 (0.57)	6.43 (0.74)	9.28 (2.95)	11.29 (1.14)
	Peak	8.49 (0.55)	8.85 (1.13)	11.31 (3.01)	14.3 (1.16)
	Fall	10.72 (0.6)	11.91 (1.63)	13.37 (2.85)	16.53 (0.94)

All values were measured from the trial onset and are expressed in seconds. Listening task: *N* = 31 for Scan 1 and Scan 2; Listening-reciting task: *N* = 31 for Scan 1 and *N* = 29 for Scan 2.

### 3.4 Temporal relationships between speech production and hemodynamic responses

The timings of speech onset and offset, together with the rise, peak, and fall of hemodynamic responses, were examined across subjects for three ICs identified for the listening-reciting task (Fig. 8; Tables 2 and 3). In addition to the median and interquartile range (IQR), the mean and standard deviation of the peak timing of each IC’s hemodynamic response were compared with the onset of speech output (6.15 ± 0.34 s and 5.98 ± 0.28 s for Scan 1 and Scan 2, respectively).

**Table 3.** Medians and interquartile ranges (IQR) of key timings of speech output and hemodynamic responses in the IC time courses (listening-reciting task).

	Speech		Hemodynamic response			
	Onset	Offset	IC	STC	IFG	SMC
Scan 1	6.15 (0.34)	10.75 (0.87)	Rise	6.61 (0.93)	8.48 (3.62)	10.59 (1.35)
			Peak	8.92 (0.77)	11.72 (5.06)	13.61 (1.87)
			Fall	11.52 (1.66)	14.4 (4.1)	16.39 (1.45)
Scan 2	5.98 (0.28)	10.67 (1.08)	Rise	6.32 (0.92)	7.93 (3.83)	11.05 (1.53)
			Peak	8.63 (1.02)	9.89 (4.93)	14.01 (1.45)
			Fall	11.42 (1.98)	12.61 (5.13)	16.31 (1.26)

All values were measured from the trial onset and are expressed in seconds. *N* = 31 for Scan 1 and *N* = 29 for Scan 2.

For the STC IC, the mean times of the hemodynamic response peak were 9.21 ± 1.2 s (Scan 1) and 8.85 ± 1.13 s (Scan 2) from trial onset, with average lags of 3.06 s and 2.87 s after the onset of speech output for Scan 1 and Scan 2, respectively. The mean times for the IFG IC to reach peak from trial onset occurred later at 11.64 ± 2.68 s (Scan 1) and 11.31 ± 3.01 s (Scan 2), with average lags of 5.49 s and 5.33 s after the speech onset for Scan 1 and Scan 2, respectively. The hemodynamic response of the SMC IC peaked at 14.06 ± 1.11 s (Scan 1) and 14.3 ± 1.16 s (Scan 2) from trial onset, with the longest post-speech-onset lags of 7.91 s and 8.32 s for Scan 1 and Scan 2, respectively.

The temporal relationships between speech onset and the rise of the hemodynamic responses in the STC IC display the most tightly clustered distributions in both scans, followed by the SMC IC with slightly wider distributions, and the IFG IC with highly dispersed distributions (open circles in Fig. 8; see also IQR values in Table 3). The temporal relationships between speech offset and the fall of the hemodynamic responses in the SMC IC display the most concentrated distributions in both scans, while the STC and IFG ICs show dispersed distributions (open squares in Fig. 8; see also IQR values in Table 3).

Overall, ICs in the auditory and sensorimotor cortices, compared with cognitive processing regions in the frontal cortex, were more predictable due to the fixed timings of the stimulus phase (0–5 s) and production phase (5–10 s) within each trial (see Section 4).

## 4 Discussion

Since the 1990s, fMRI has been one of the noninvasive neuroimaging techniques used for investigating the neural basis of cognition. Although fMRI offers high spatial resolution for mapping brain functions, the rapid switching of gradient magnetic fields during echo planar imaging generates loud noise, posing a major challenge for fMRI investigations of the auditory system and speech processing. For an experiment to successfully detect the neural response to a delivered auditory stimulus, that response must be sufficiently robust to rise above the competing neural activity evoked by the scanner noise itself (Dewey et al., 2021).

To mitigate the interference due to scanner noise, sparse-sampling fMRI designs have been developed and used to study speech processing (Erb et al., 2013; Golfinopoulos et al., 2011; Hervais-Adelman et al., 2015; Longcamp et al., 2019; Murray et al., 2022; Orellana et al., 2014; Perrachione & Ghosh, 2013; Tao et al., 2026). While effective at creating quiet periods for stimulus presentation and over speech production, this approach imposes significant constraints on experimental designs and analyses. Prolonged trial durations are required to accommodate both the stimulus presentation and the subsequent lag of the hemodynamic response, all while ensuring sufficient data is collected for statistical power. For instance, in a sound mimicry study that integrated both perception and production within a single trial, sparse-sampling designs with an interval of 10 s would require a long scan time to obtain sufficient trials for statistical analysis (Lewis et al., 2018). Furthermore, the significant data loss due to sparse sampling makes it unable to characterize the full profiles of hemodynamic responses during complex, multi-stage tasks like auditory perception followed by overt production.

In addition to acoustic noise, head motion artifacts remain a significant source of variance in fMRI data, capable of introducing spurious correlations and obscuring genuine neural signals (Liu, 2016). In the present study, we implemented a comprehensive strategy to minimize head motion throughout the functional session, allowing for tasks involving the overt production of 5-s sentences. While the efficacy of individual methods, like custom-molded headcases or masks (Jolly et al., 2020; Power et al., 2019), can be debated, our approach combined several practices: (1) subjects underwent a training session with real-time head motion feedback to train them to remain still in a mock scanner; (2) subjects wore custom-molded thermoplastic masks for rigid head stabilization; and (3) the head motion was further confined by filling residual air gaps within the head coil. This concerted effort effectively restricted the range of head displacement to within 1 mm and rotation to below 1° per scan in all participants, providing a stable foundation for the high-resolution analyses to follow.

Through targeted technical efforts to mitigate the challenges of scanner noise and head motion during speech perception and overt production, we demonstrated improved experimental protocols for continuous sampling with real-time auditory feedback. These technical solutions enable us to address advanced questions in the perception and overt production of sentences that are considerably more difficult to investigate with sparse-sampling designs. In an event-related fMRI experiment, as long as two auditory stimuli (events) are separated by a prolonged interval, a peak of the hemodynamic response to each stimulus can be distinctly identified from the BOLD time course (Hickok et al., 2003). However, the hemodynamic responses to two stimuli would not be easily separated when they are presented in close succession.

In this study, we explored the use of ICA for unraveling independent brain processes associated with sentence-level speech perception, planning, and production. Each functional scan was subjected to ICA, and the resulting IC maps were displayed on the cortical surfaces of an individual subject. We then selected IC maps with similar activation patterns across subjects by visual inspection and combined them through surface-to-surface registration, yielding group-average IC maps on the common cortical surface of *fsaverage* (Fischl et al., 1999; Sereno et al., 2022). The spatial reliability of activation patterns in each group-average IC map was assessed by a surface-based t-test. Each group-average IC map obtained through manual annotation served as an initial model for assessing map similarity across subjects. An updated group-average IC map may be computed after removing individual IC maps that exhibited low correlations with the initial model (e.g., IC maps with *r* < 0.2 in Supplementary Tables S4, S5).

The surface-based clustering of individual IC maps proposed in the current study is fundamentally different from the volume-based group-ICA method (e.g., Janssen & Mendieta, 2020), where the functional volumes were combined across subjects before being subjected to ICA. Our method yields subject-level IC maps and time courses with high spatial and temporal precision, enabling individualized mapping and detection of outliers. As shown in Figures 3–6 (E) the overall waveforms of IC time courses are consistent across most subjects, while the key timings of hemodynamic responses vary (Fig. 8). Although most subjects displayed typical spatial activation patterns and periodic signals, we could not identify the reasons (e.g., head motion or poor task performance) for the irregular time courses from two subjects that had normal IC maps (Figs. 4–6, E, Scan 2). It is likely that these irregular time courses arise from the limitations of the current ICA algorithm, which failed to converge and yielded incomplete decompositions for these two subjects. In summary, we demonstrated a data processing pipeline for surface-based IC selection across individual subjects and computed group-average IC maps to construct baseline models. We believe these initial procedures and models could be expanded into a comprehensive framework for fully automatic classification of IC maps and time courses in future studies.

In the current study, we selected three primary clusters of IC maps with the largest and highest activations concentrated in the superior temporal cortex, inferior frontal gyrus, and sensorimotor cortex. While additional smaller activations were present in each cluster, the resulting surface-based group-average IC maps were named according to the most dominant activation regions: STC, IFG, and SMC. The time courses of these ICs exhibited sequential activations corresponding to the speech perception (STC), articulatory planning (IFG) (Basilakos et al., 2018), and articulation (SMC) phases during the listening-reciting task (Figs. 4–6).

While the spatial ICA method used in the current study did not resolve separate ICs associated with the processing of external speech and self-generated speech, it revealed that the similar STC ICs identified for the listening and listening-reciting tasks exhibited different activation profiles. Compared with the listening task, the listening-reciting task induced a wider profile of hemodynamic response in the STC IC (Fig. 7B). With a continuous sampling rate of 1 TR/s, we were able to upsample and contrast the IC time courses between the listening-reciting and listening tasks. Based on the properties of a linear system (Boynton et al., 2012), we postulated that the widened profile resulted from the linear superposition of two hemodynamic responses – one to external speech during the perception phase (0–5 s) and the second to online monitoring of self-generated speech during the production phase (5–10 s). Subtracting the STC IC time course of the listening task from that of the listening-reciting task indeed revealed a second hemodynamic response (Fig. 7B), with its peak timing aligned to that of the SMC IC. In some subjects, the second hemodynamic response to self-generated speech appeared as a small hump embedded in the descending section of the widened hemodynamic profile in the STC IC (Fig. 4E).

In addition to ICA decomposition, continuous sampling enables the examination of the temporal relationships between sentence-level speech production and the evoked hemodynamic responses with high precision. The hemodynamic profiles of the STC IC (listening task) and the SMC IC (listening-reciting task) represent the BOLD responses to the external stimulus and speech output, respectively. For the listening task, the peak of the STC IC time course occurred ~8.5 s after the onset of a spoken sentence (external speech) (Fig. 7B, Tables 1 and 2). For the listening-reciting task, the peak of the SMC IC time course occurred ~8 s after the onset of speech output at ~6 s within a 16-s trial (Fig. 7A, Table 3). These peak latencies, which are later than the 5 to 6 s range found in typical hemodynamic responses, indicate extended processing time for sentence-level stimuli. Furthermore, the peak latencies in the STC and SMC ICs were less dispersed than those in the IFG IC, suggesting stable hemodynamic responses in the auditory and sensorimotor cortices across subjects due to the well-defined timings of the stimulus and response phases.

The hemodynamic response profiles of the IFG IC may represent the speech rehearsing and articulatory planning processes and do not directly align with the timing of a stimulus or response. Indeed, the IFG IC time courses exhibited large inter-subject variability in the duration between the speech onset and the hemodynamic peak (Fig. 8, Table 3), suggesting differential strategies in rehearsal (e.g., some subjects may start articulatory planning as soon as the stimulus onset occurs, while others may only start after a whole sentence has been heard). This variability underscores a critical limitation of sparse-sampling designs. As the typical window for image acquisition is limited to 2 s per trial (e.g., Hervais-Adelman et al., 2015; Perrachione & Ghosh, 2013; Tao et al., 2026), sparse-sampling fMRI cannot reliably capture the temporally variable responses to high-level language processing in frontal regions. Future sparse-sampling designs involving sentence-level stimuli could therefore incorporate longer and more flexible acquisition windows, informed by the temporal profiles revealed through continuous sampling approaches.

While informative, this study entails some limitations. First, our listening-reciting task was designed to enable precise control over speech onset and provide well-defined perception and production phases. However, the listening-reciting task does not fully capture the cognitive demands of natural, generative speech production; rather, it relies predominantly on the phonological loop component of working memory (Baddeley, 2003). Consequently, the neural dynamics we observed, particularly in the inferior frontal gyrus, may reflect the articulatory planning of rehearsal to a greater extent than a higher-level linguistic formulation. Nonetheless, the controlled nature of this task served as a critical first step in validating the feasibility of continuous sampling and in disentangling temporally overlapping hemodynamic responses. Second, variances in bilingual control could be present as the tasks were completed in a second language (Tang et al., 2023). Although such variances in bilingual control are much more likely to affect domain-general brain regions, such as the IFG, rather than sensory and motor regions, such as the STC and SMC (Tao et al., 2021). Future studies could investigate whether the results are generalizable to native language speakers and monolinguals.

Finally, the spatial ICA here did not yield separate independent components associated with the processing of external speech versus self-generated speech. This likely reflects the overlapping roles in the superior temporal cortex that subserve the perception of external speech, auditory-motor transformation (Hickok et al., 2009, 2011; Pa & Hickok, 2008), and self-monitoring of one’s own vocal output (Tourville & Guenther, 2011). Such overlapping functional networks may not be resolvable by ICA at the spatial and temporal resolution of conventional fMRI, highlighting the need for future studies to employ complementary techniques, such as multivoxel spatiotemporal pattern analysis, to more effectively dissociate these mixed signals.

In conclusion, we present methodological advancements that facilitate continuous fMRI sampling during sentence-level speech perception and production tasks. Independent component analysis revealed time-resolved hemodynamic responses across multiple ICs associated with the perception, planning, production, auditory-motor transformation, and self-monitoring processes. The temporal information identified, such as the delay between the speech onset and the hemodynamic peak, may facilitate future auditory fMRI studies in determining the optimal timings for stimulus presentation, overt production, and image acquisition for either continuous or sparse sampling designs. This study not only demonstrates the feasibility of naturalistic speech tasks but also lays the groundwork for future research into tasks like simultaneous interpreting and collaborative singing, and explores the nuanced interactions between externally perceived auditory input and awareness of internally generated sounds, advancing our knowledge of cognitive processing in these multifaceted activities.

## Supplementary Material

Supplementary Material

## Data Availability

The raw MRI data containing skull and facial features of individual subjects cannot be made openly available due to ethical restrictions regarding participant confidentiality. Derived fMRI data for creating surface-based IC maps from anonymous subjects and group average are included in the *Huang and Sereno Brain Atlas*, openly available on the Open Science Framework (https://osf.io/b2puq/). Custom codes for analyzing volume-based and surface-based fMRI data are included in *csurf* (a FreeSurfer-compatible package, https://pages.ucsd.edu/~msereno/csurf/).
