# Supplementary material for: Time-resolved hemodynamic responses to sentence-level speech perception, production, and self-monitoring
Source: Imaging Neurosci (Camb). 2026 Sep 21;4:IMAG.a.1375. doi: 10.1162/IMAG.a.1375 (PMC13595370; doi:10.1162/IMAG.a.1375)
Supplement: Supplementary file 1 [file IMAG.a.1375_supp.pdf]

## Supplementary Materials

# Time-resolved hemodynamic responses to sentence-level speech perception, production, and self-monitoring

Teng Ieng Leong[1,2†], Aotong Li[1,3], Jian Hwee Ang[1,3], Barry Lee Reynolds[1,4], Yan Tian[1,2], Cheok Teng Leong[1,3], Chi Un Choi[1], Martin I. Sereno[5], Defeng Li[1,2], Victoria Lai Cheng Lei[1,2*], Ruey-Song Huang[1,3†*]

[1]Centre for Cognitive and Brain Sciences, University of Macau, Taipa, Macau SAR, China
[2]Faculty of Arts and Humanities, University of Macau, Taipa, Macau SAR, China
[3]Faculty of Science and Technology, University of Macau, Taipa, Macau SAR, China
[4]Faculty of Education, University of Macau, Taipa, Macau SAR, China
[5]Department of Cognitive Science, University of California, San Diego, La Jolla, CA, 92093-0515, USA

[†]These authors contributed equally to this work.

***Corresponding authors:**
Ruey-Song Huang (rueysong.huang@gmail.com), Victoria Lai Cheng Lei (viclcl@um.edu.mo)

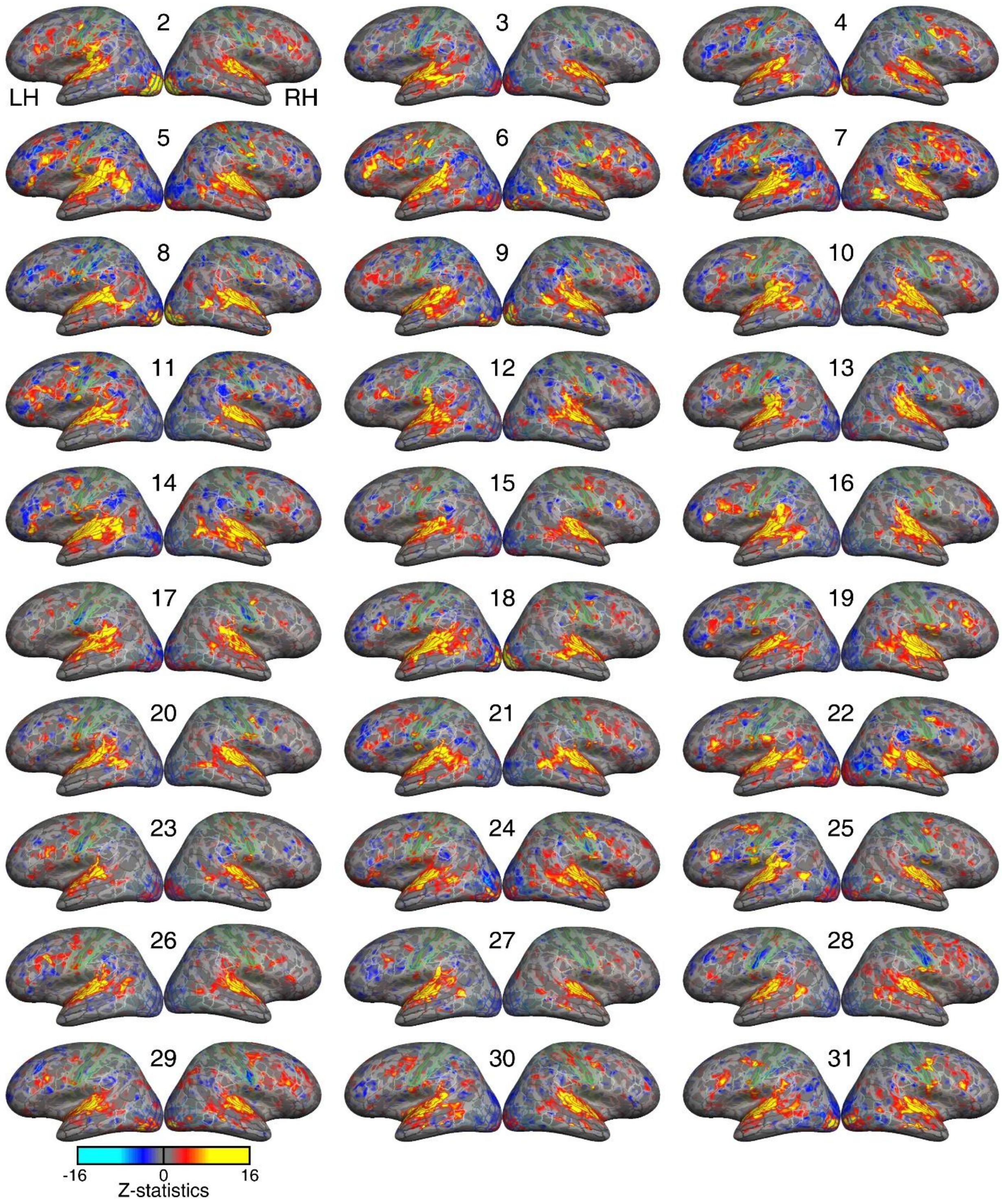


**Supplementary Figure 1.** Individual spatial maps (Subjects 2-31) of the STC IC in Scan 1 of the listening task. See Subject 1 in Fig. 3A. LH: left hemisphere; RH: right hemisphere.

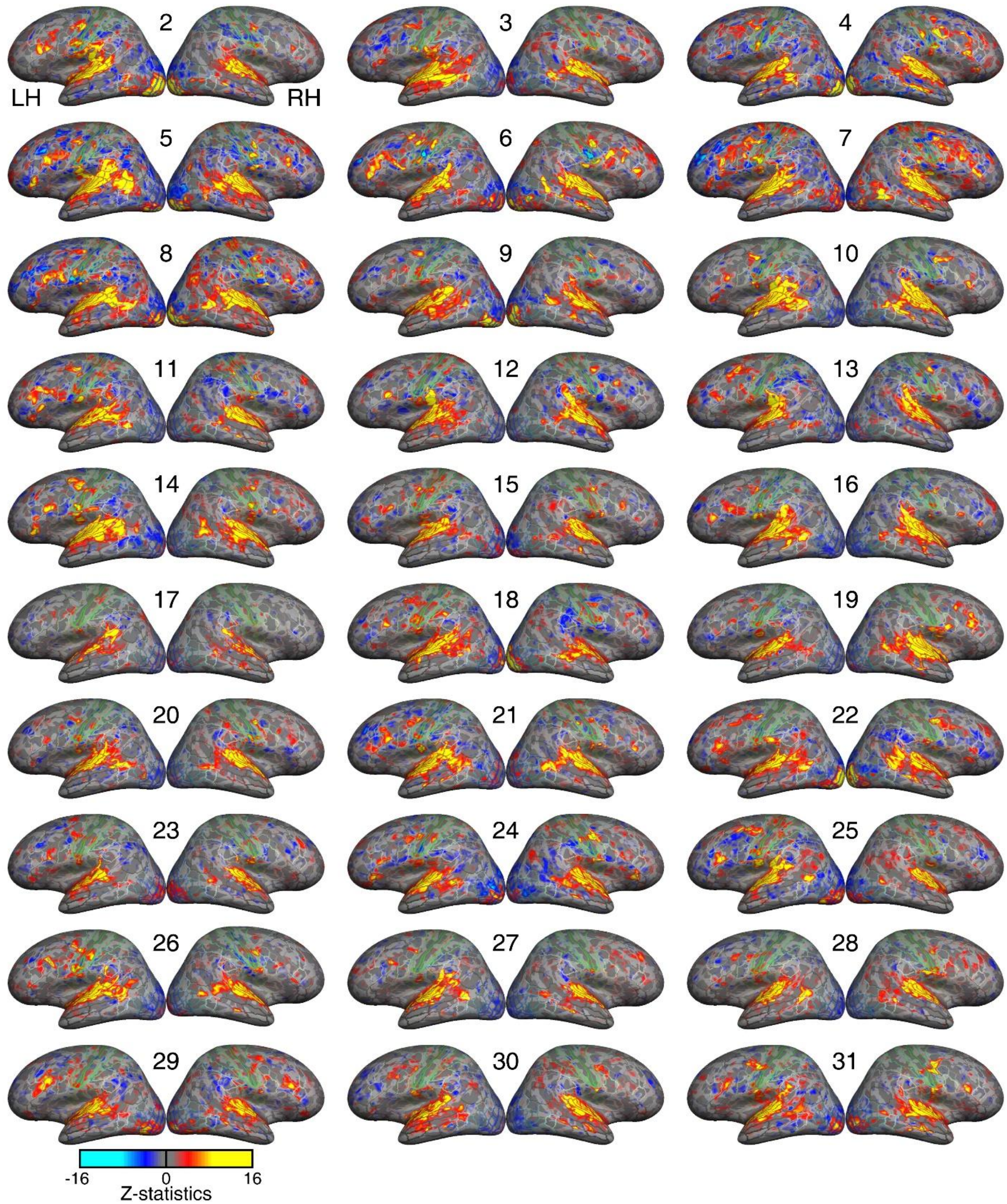


**Supplementary Figure 2.** Individual spatial maps (Subjects 2-31) of the <u>STC IC in Scan 2 of the listening task</u>. See Subject 1 in Fig. 3A. LH: left hemisphere; RH: right hemisphere.

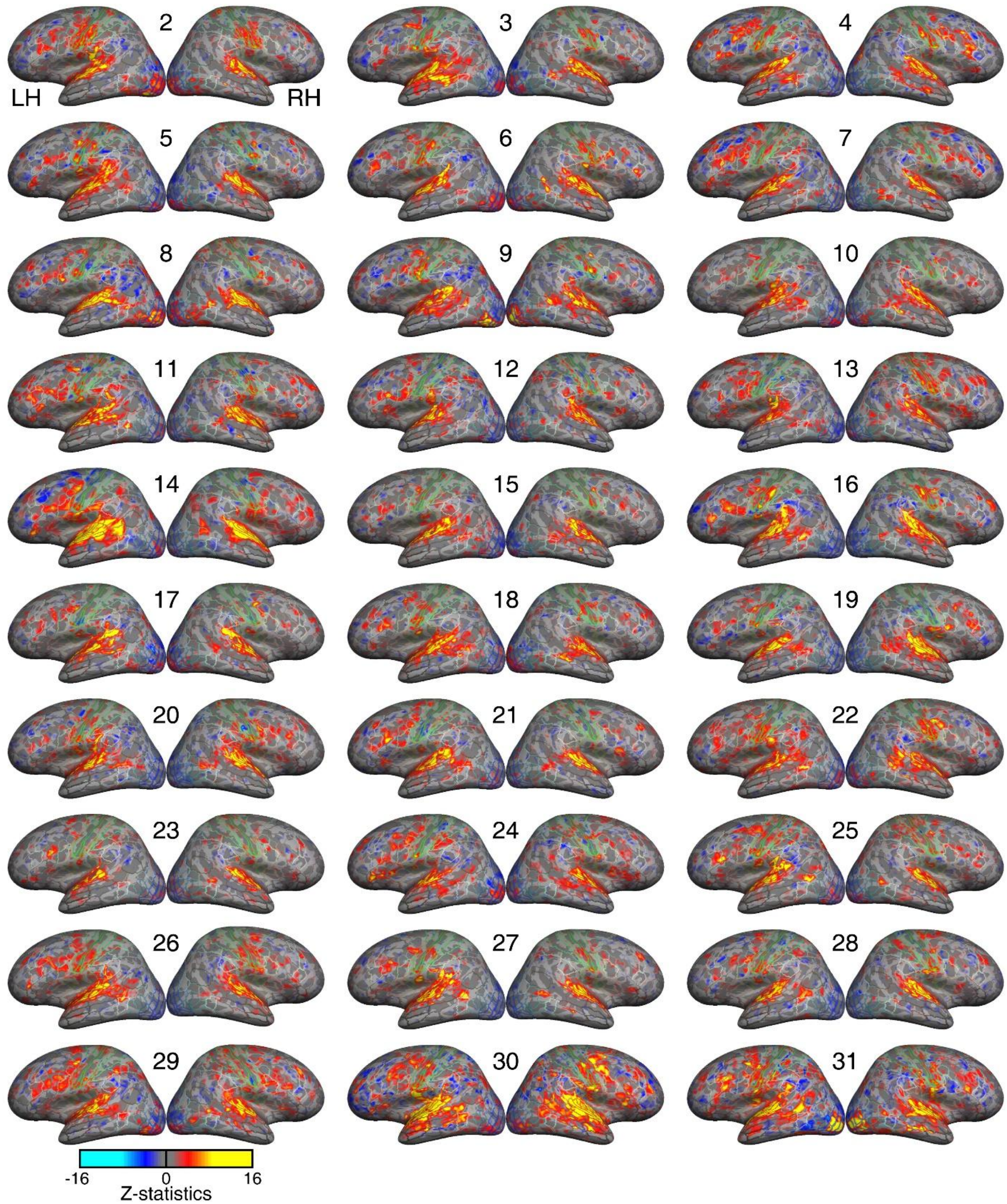


**Supplementary Figure 3.** Individual spatial maps (Subjects 2-31) of the STC IC in Scan 1 of the listening-reciting task. See Subject 1 in Fig. 4A. LH: left hemisphere; RH: right hemisphere.

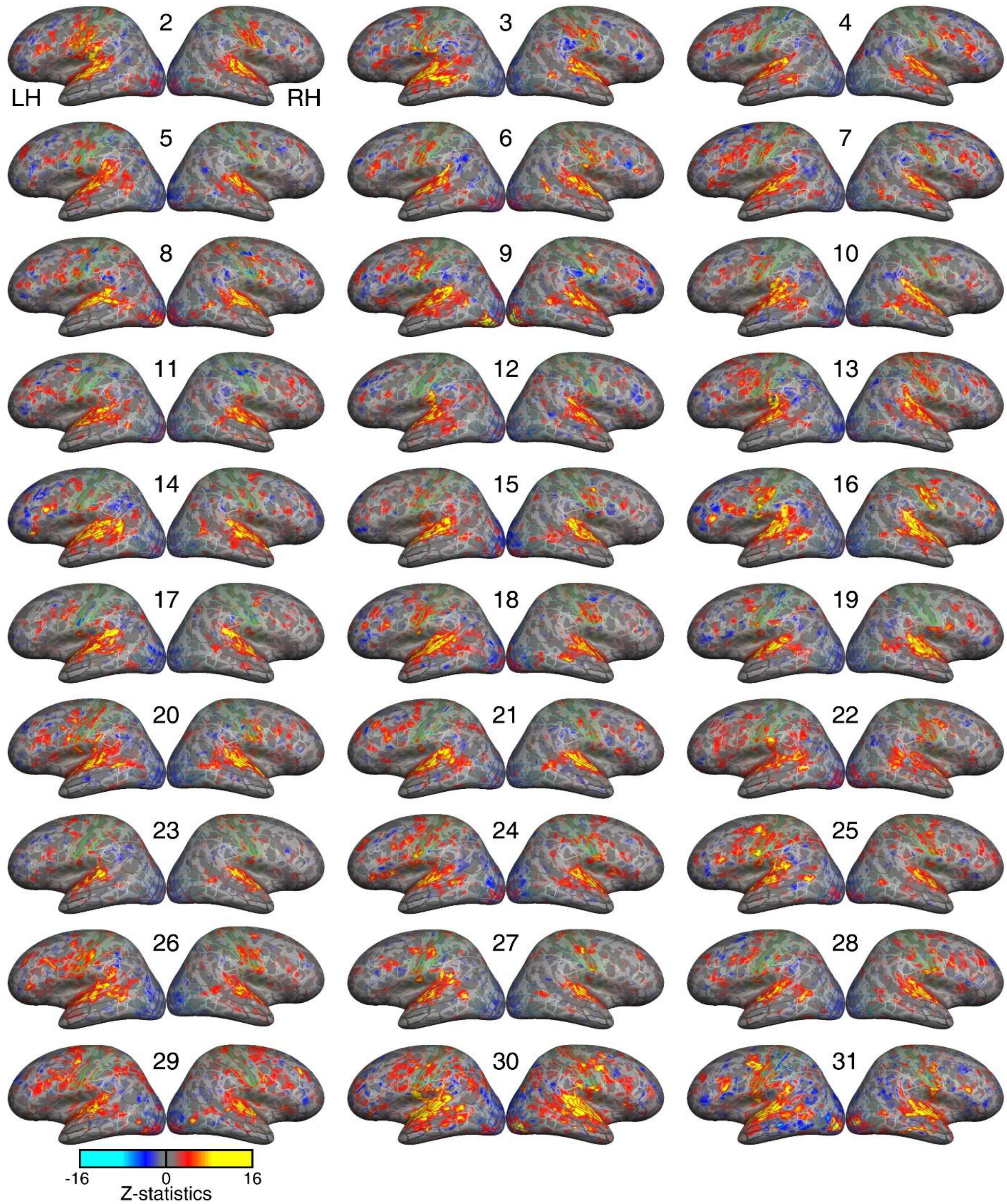


**Supplementary Figure 4.** Individual spatial maps (Subjects 2-31) of the <u>STC IC in Scan 2 of the listening-reciting task</u>. See Subject 1 in Fig. 4A. LH: left hemisphere; RH: right hemisphere.

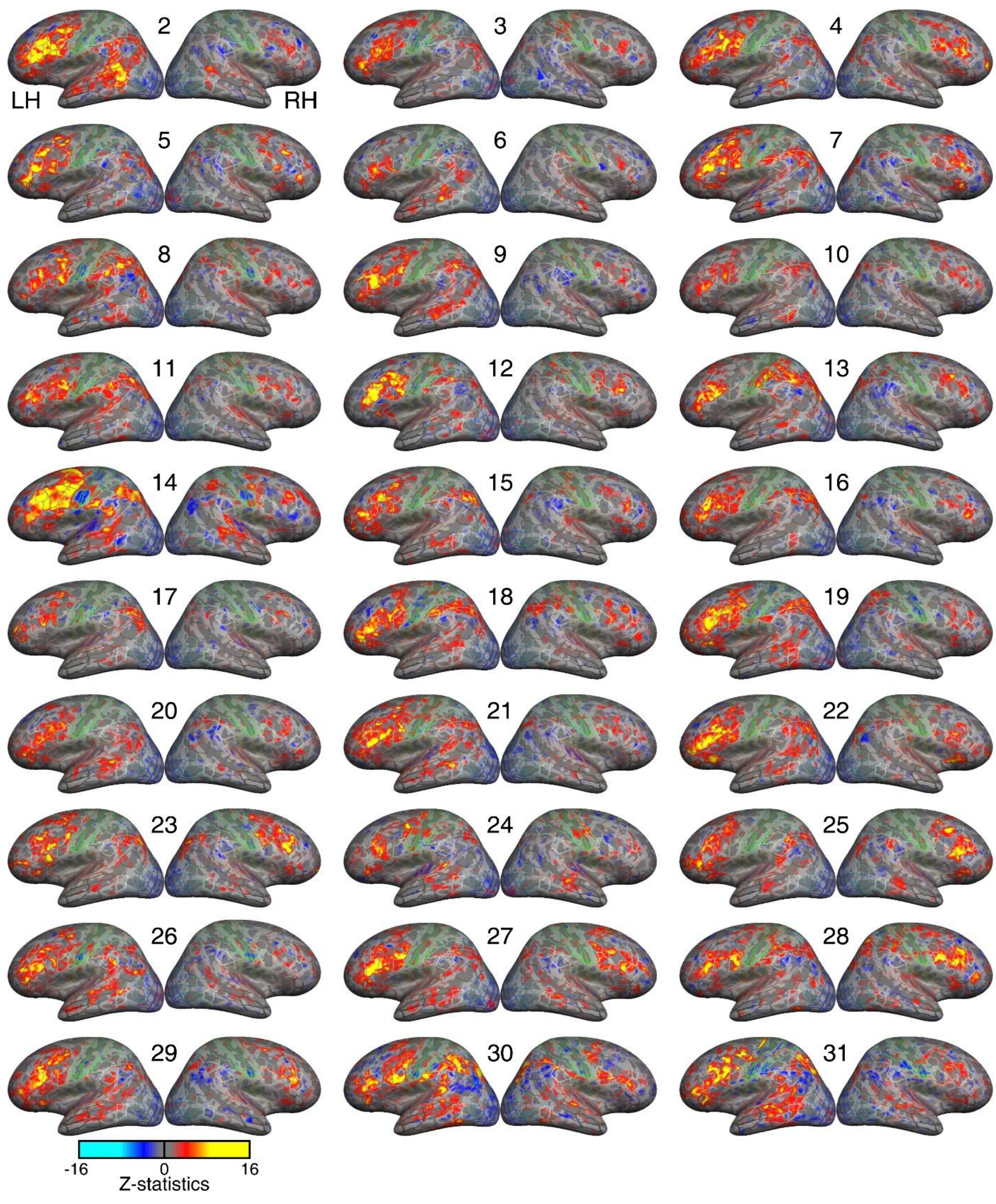


**Supplementary Figure 5.** Individual spatial maps (Subjects 2-31) of the IFG IC in Scan 1 of the listening-reciting task. See Subject 1 in Fig. 5A. LH: left hemisphere; RH: right hemisphere.

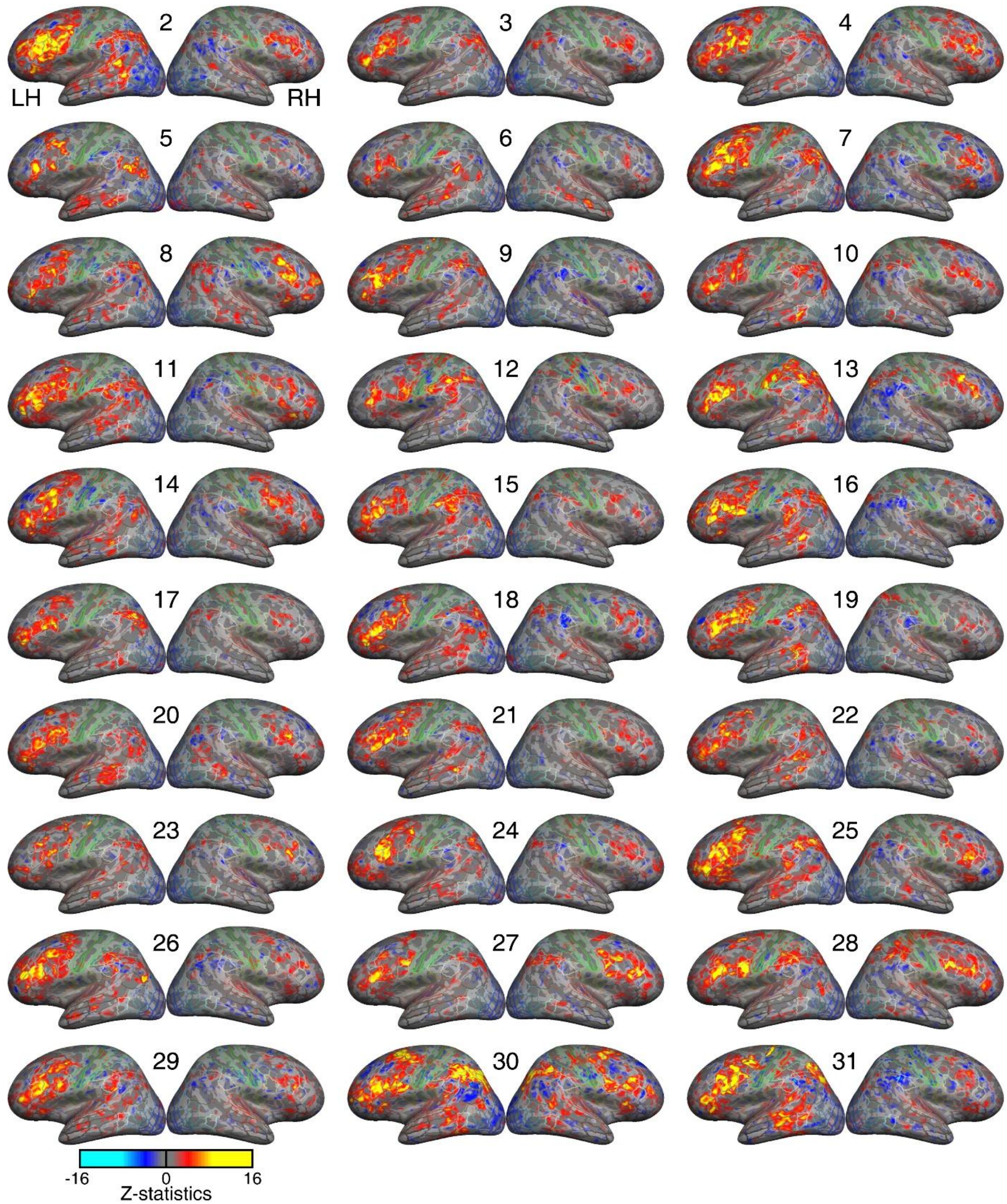


**Supplementary Figure 6.** Individual spatial maps (Subjects 2-31) of the IFG IC in Scan 2 of the listening-reciting task. See Subject 1 in Fig. 5A. LH: left hemisphere; RH: right hemisphere.

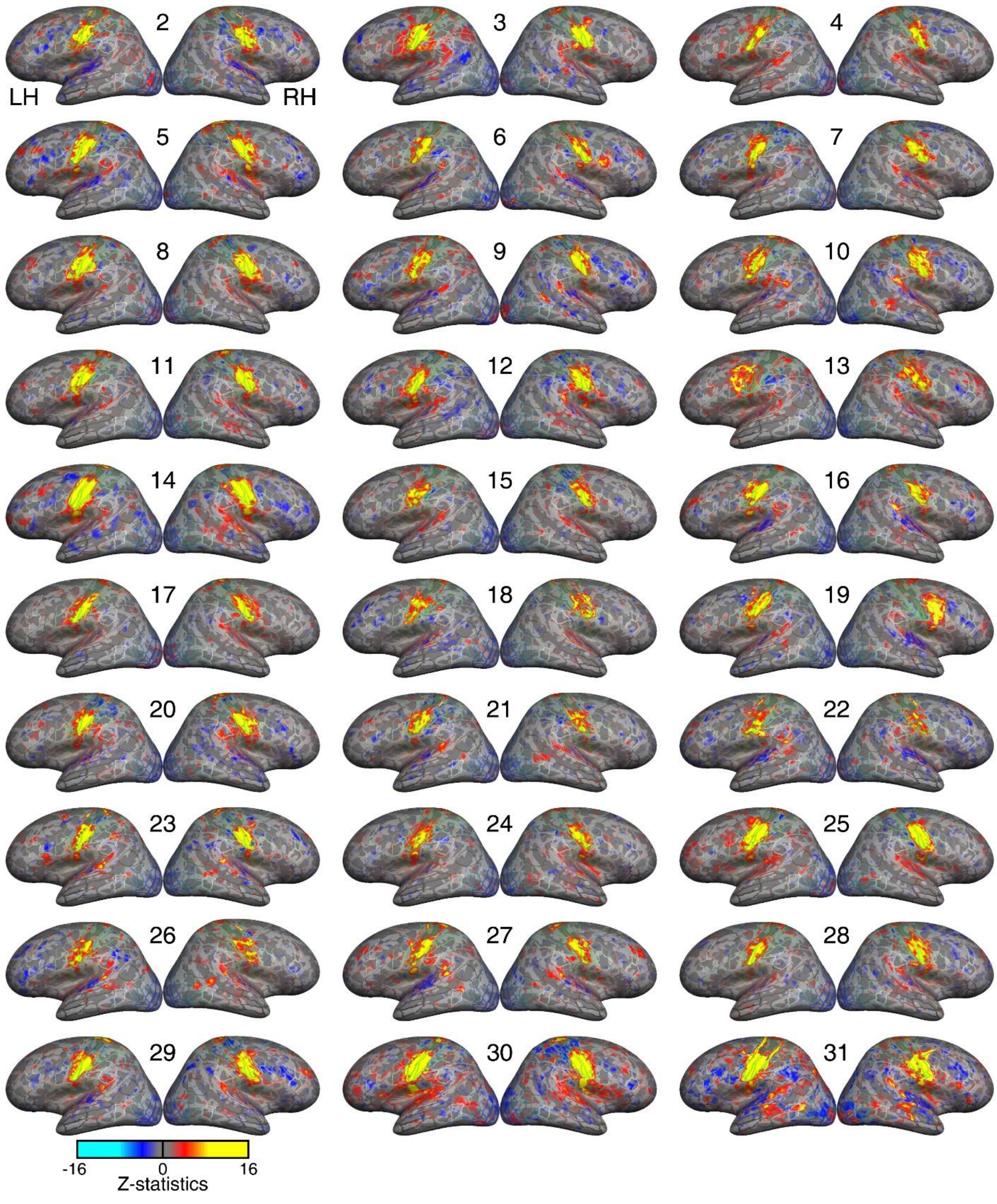


**Supplementary Figure 7.** Individual spatial maps (Subjects 2-31) of the SMC IC in Scan 1 of the listening-reciting task. See Subject 1 in Fig. 6A. LH: left hemisphere; RH: right hemisphere.

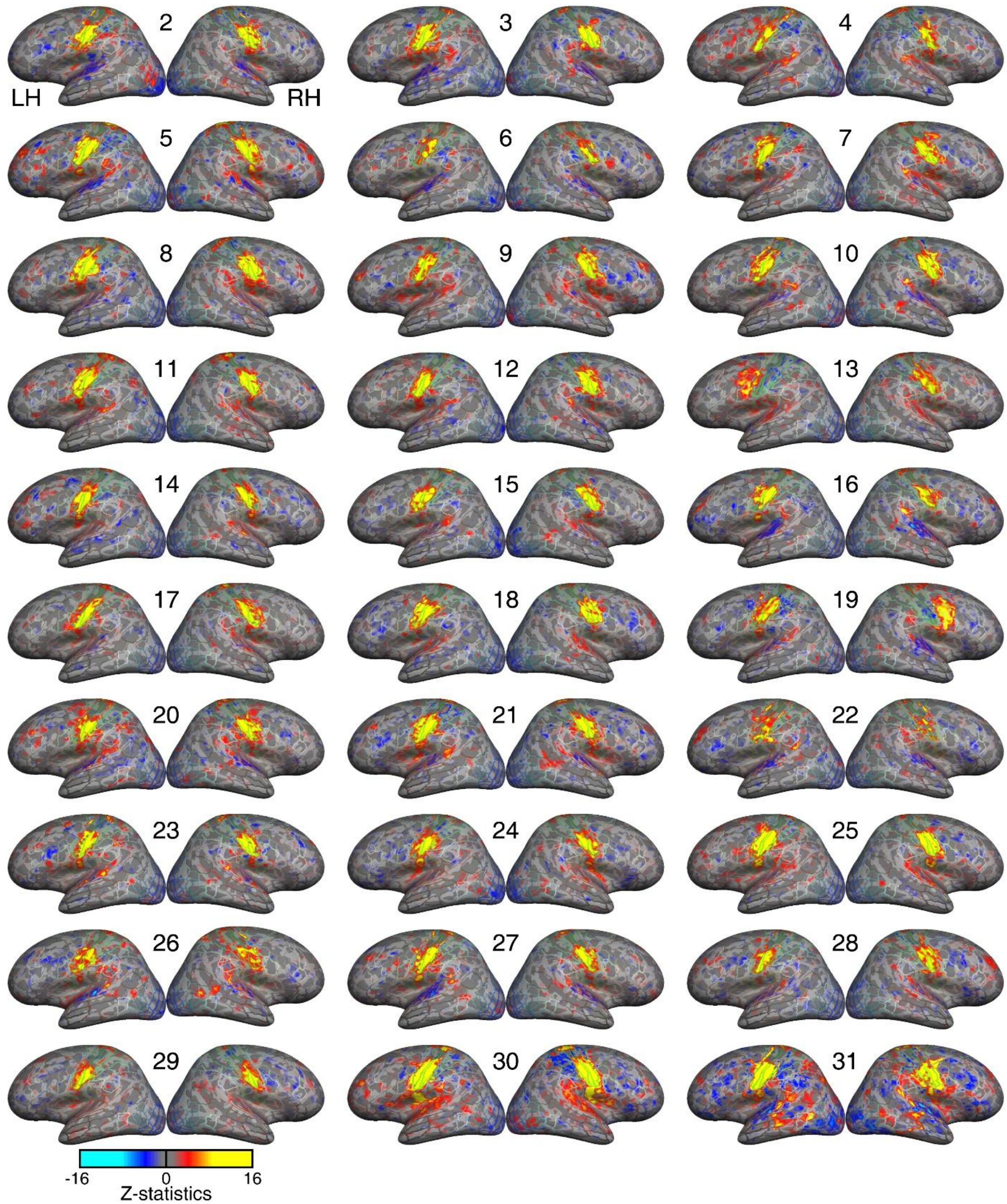


**Supplementary Figure 8.** Individual spatial maps (Subjects 2-31) of the SMC IC in Scan 2 of the listening-reciting task. See Subject 1 in Fig. 6A. LH: left hemisphere; RH: right hemisphere.

**Supplementary Table 1.** Correlation coefficients between the IC spatial maps of Scan 1 and Scan 2. For each hemisphere of each subject, a correlation coefficient is computed by the function *Corr* (Scan 1 map, Scan 2 map) in MATLAB. LH: left hemisphere; RH: right hemisphere. Bold numbers indicate low correlation ($r < 0.2$).

| Task/IC | Listening: STC IC maps in Fig. 3A (Subject 1); Supplementary Figures 1 and 2 (Subjects 2-31) | | Listening-reciting: STC IC maps in Fig. 4A (Subject 1); Supplementary Figures 3 and 4 (Subjects 2-31) | | Listening-reciting: IFG IC maps in Fig. 5A (Subject 1); Supplementary Figures 5 and 6 (Subjects 2-31) | | Listening-reciting: SMC IC maps in Fig. 6A (Subject 1); Supplementary Figures 7 and 8 (Subjects 2-31) | |
|---|---|---|---|---|---|---|---|---|
| Subject | LH | RH | LH | RH | LH | RH | LH | RH |
| 1 | 0.8955 | 0.884 | 0.8647 | 0.8778 | 0.6005 | 0.494 | 0.8224 | 0.8661 |
| 2 | 0.9144 | 0.8823 | 0.8523 | 0.8284 | 0.7844 | 0.4701 | 0.8379 | 0.8005 |
| 3 | 0.8562 | 0.7828 | 0.8443 | 0.7548 | 0.6155 | 0.2257 | 0.8345 | 0.8561 |
| 4 | 0.8613 | 0.8829 | 0.8432 | 0.8187 | 0.6988 | 0.5427 | 0.7722 | 0.7582 |
| 5 | 0.8708 | 0.8681 | 0.7688 | 0.7439 | 0.2699 | **0.0745** | 0.8885 | 0.8932 |
| 6 | 0.89 | 0.906 | 0.849 | 0.8602 | 0.5294 | 0.2997 | 0.7203 | 0.7488 |
| 7 | 0.9011 | 0.9152 | 0.7817 | 0.7462 | 0.7194 | 0.6491 | 0.8053 | 0.7755 |
| 8 | 0.8972 | 0.9183 | 0.847 | 0.858 | 0.4652 | **0.1731** | 0.8652 | 0.854 |
| 9 | 0.802 | 0.8817 | 0.8328 | 0.8169 | 0.7374 | 0.3348 | 0.6062 | 0.6921 |
| 10 | 0.8701 | 0.893 | 0.7946 | 0.7688 | 0.6446 | 0.4994 | 0.8126 | 0.8211 |
| 11 | 0.9087 | 0.8715 | 0.8299 | 0.7829 | 0.6829 | 0.3254 | 0.8075 | 0.8342 |
| 12 | 0.7956 | 0.8057 | 0.5712 | 0.613 | 0.3843 | **0.1565** | 0.6978 | 0.791 |
| 13 | 0.8871 | 0.8344 | 0.7228 | 0.7171 | 0.7596 | 0.5322 | 0.455 | 0.5934 |
| 14 | 0.9241 | 0.8533 | 0.7917 | 0.7175 | 0.4106 | 0.2546 | 0.725 | 0.7243 |
| 15 | 0.8666 | 0.8674 | 0.8201 | 0.8317 | 0.4756 | 0.2462 | 0.7262 | 0.7654 |
| 16 | 0.9013 | 0.9016 | 0.8743 | 0.8849 | 0.6769 | 0.3384 | 0.8314 | 0.809 |
| 17 | 0.7186 | 0.6618 | 0.8605 | 0.8125 | 0.5139 | **0.1185** | 0.8365 | 0.8514 |
| 18 | 0.8515 | 0.791 | 0.8189 | 0.7703 | 0.6953 | 0.5058 | 0.6386 | 0.6077 |
| 19 | 0.8999 | 0.9369 | 0.8789 | 0.8849 | 0.7652 | 0.4843 | 0.7459 | 0.7653 |
| 20 | 0.8873 | 0.8895 | 0.8179 | 0.8417 | 0.7009 | 0.3102 | 0.7672 | 0.8102 |
| 21 | 0.894 | 0.8954 | 0.8377 | 0.8251 | 0.7592 | 0.4688 | 0.7531 | 0.7314 |
| 22 | 0.8815 | 0.8475 | 0.7885 | 0.7898 | 0.6398 | 0.3137 | 0.7916 | 0.7439 |
| 23 | 0.8518 | 0.8356 | 0.7768 | 0.7768 | 0.6947 | 0.6258 | 0.8927 | 0.8797 |
| 24 | 0.8598 | 0.8135 | 0.7508 | 0.7647 | 0.483 | **0.1449** | 0.6212 | 0.6639 |
| 25 | 0.8872 | 0.8853 | 0.8275 | 0.7996 | 0.7097 | 0.5324 | 0.8028 | 0.8025 |
| 26 | 0.8617 | 0.7499 | 0.8127 | 0.7067 | 0.6164 | 0.3632 | 0.7384 | 0.7448 |
| 27 | 0.905 | 0.852 | 0.8262 | 0.7469 | 0.6463 | 0.6114 | 0.8142 | 0.7873 |
| 28 | 0.8339 | 0.8817 | 0.7197 | 0.7559 | 0.6478 | 0.661 | 0.6762 | 0.7494 |
| 29 | 0.8634 | 0.8423 | 0.8218 | 0.812 | 0.6963 | 0.4575 | 0.662 | 0.7356 |
| 30 | 0.8473 | 0.8682 | 0.8483 | 0.8866 | 0.6079 | 0.4419 | 0.8782 | 0.869 |
| 31 | 0.8916 | 0.8663 | 0.7963 | 0.7961 | 0.7698 | 0.4144 | 0.896 | 0.8941 |

**Supplementary Table 2.** Correlation coefficients between single-subject maps and the group-average map of the STC IC cluster for Scan 1 and Scan 2 of the listening task. For each hemisphere in each subject, a correlation coefficient is computed by the function *Corr* (Single-subject map, Group-average map) in MATLAB. LH: left hemisphere; RH: right hemisphere. Bold numbers indicate low correlation ($r < 0.2$).

| Scan | Scan 1: Subject 1 (Fig. 3A) and Subjects 2-31 (Suppl. Fig. 1) vs. Group-average (Fig. 3B) | | Scan 2: Subject 1 (Fig. 3A) and Subjects 2-31 (Suppl. Fig. 2) vs. Group-average (Fig. 3B) | |
|---|---|---|---|---|
| Subject | LH | RH | LH | RH |
| 1 | 0.7699 | 0.7559 | 0.7696 | 0.7754 |
| 2 | 0.686 | 0.6869 | 0.7362 | 0.7585 |
| 3 | 0.6467 | 0.6693 | 0.7446 | 0.7748 |
| 4 | 0.7163 | 0.7594 | 0.712 | 0.7554 |
| 5 | 0.7935 | 0.8186 | 0.7833 | 0.8125 |
| 6 | 0.7286 | 0.7667 | 0.7219 | 0.7707 |
| 7 | 0.8014 | 0.7583 | 0.8111 | 0.7572 |
| 8 | 0.7696 | 0.7441 | 0.7732 | 0.7669 |
| 9 | 0.6967 | 0.7426 | 0.7019 | 0.7467 |
| 10 | 0.7425 | 0.7316 | 0.7738 | 0.7509 |
| 11 | 0.6824 | 0.7045 | 0.6984 | 0.7258 |
| 12 | 0.6886 | 0.7039 | 0.7321 | 0.7457 |
| 13 | 0.6304 | 0.7225 | 0.6004 | 0.6526 |
| 14 | 0.8146 | 0.7535 | 0.8084 | 0.7571 |
| 15 | 0.7666 | 0.788 | 0.7797 | 0.7965 |
| 16 | 0.7614 | 0.7878 | 0.7808 | 0.7857 |
| 17 | 0.6839 | 0.6943 | 0.5062 | 0.5731 |
| 18 | 0.7922 | 0.6989 | 0.7359 | 0.5705 |
| 19 | 0.7509 | 0.8361 | 0.7463 | 0.8492 |
| 20 | 0.7882 | 0.78 | 0.7897 | 0.7836 |
| 21 | 0.8048 | 0.8348 | 0.814 | 0.8393 |
| 22 | 0.7424 | 0.6975 | 0.6939 | 0.6434 |
| 23 | 0.6764 | 0.726 | 0.6754 | 0.711 |
| 24 | 0.7246 | 0.7201 | 0.7403 | 0.6998 |
| 25 | 0.7712 | 0.7724 | 0.7197 | 0.7461 |
| 26 | 0.784 | 0.7646 | 0.7778 | 0.7393 |
| 27 | 0.7456 | 0.7565 | 0.7565 | 0.7551 |
| 28 | 0.7435 | 0.7605 | 0.774 | 0.7741 |
| 29 | 0.7383 | 0.7226 | 0.7241 | 0.722 |
| 30 | 0.7046 | 0.7599 | 0.6934 | 0.7339 |
| 31 | 0.7648 | 0.7235 | 0.7878 | 0.7287 |

**Supplementary Table 3.** Correlation coefficients between single-subject maps and the group-average map of the STC IC cluster for Scan 1 and Scan 2 of the listening-reciting task. For each hemisphere in each subject, a correlation coefficient is computed by the function *Corr* (Single-subject map, Group-average map) in MATLAB. LH: left hemisphere; RH: right hemisphere. Bold numbers indicate low correlation ($r < 0.2$).

| Scan | Scan 1: Subject 1 (Fig. 4A) and Subjects 2-31 (Suppl. Fig. 3) vs. Group-average (Fig. 4B) | | Scan 2: Subject 1 (Fig. 4A) and Subjects 2-31 (Suppl. Fig. 4) vs. Group-average (Fig. 4B) | |
|---|---|---|---|---|
| Subject | LH | RH | LH | RH |
| 1 | 0.724 | 0.7153 | 0.7407 | 0.7363 |
| 2 | 0.6718 | 0.6623 | 0.7167 | 0.7396 |
| 3 | 0.6932 | 0.7142 | 0.6852 | 0.7028 |
| 4 | 0.666 | 0.6854 | 0.6326 | 0.6628 |
| 5 | 0.7087 | 0.7157 | 0.6724 | 0.6909 |
| 6 | 0.6608 | 0.7158 | 0.6428 | 0.7058 |
| 7 | 0.6442 | 0.6474 | 0.6774 | 0.6449 |
| 8 | 0.6699 | 0.696 | 0.6648 | 0.6855 |
| 9 | 0.6197 | 0.6554 | 0.64 | 0.6604 |
| 10 | 0.6909 | 0.6654 | 0.7119 | 0.6975 |
| 11 | 0.626 | 0.6435 | 0.6137 | 0.5714 |
| 12 | 0.5701 | 0.5904 | 0.5808 | 0.6011 |
| 13 | 0.4976 | 0.5063 | 0.5867 | 0.5853 |
| 14 | 0.7815 | 0.7317 | 0.7381 | 0.6712 |
| 15 | 0.6907 | 0.7032 | 0.6986 | 0.7461 |
| 16 | 0.6991 | 0.7278 | 0.7231 | 0.7523 |
| 17 | 0.6217 | 0.6426 | 0.6326 | 0.6163 |
| 18 | 0.7022 | 0.6462 | 0.7386 | 0.6883 |
| 19 | 0.7297 | 0.7655 | 0.7007 | 0.749 |
| 20 | 0.6609 | 0.7001 | 0.6924 | 0.7036 |
| 21 | 0.7178 | 0.7338 | 0.7179 | 0.7551 |
| 22 | 0.6179 | 0.5772 | 0.5408 | 0.4699 |
| 23 | 0.5975 | 0.6296 | 0.5873 | 0.6528 |
| 24 | 0.561 | 0.5992 | 0.648 | 0.6779 |
| 25 | 0.6719 | 0.6611 | 0.6536 | 0.6572 |
| 26 | 0.7288 | 0.6468 | 0.6881 | 0.6332 |
| 27 | 0.7154 | 0.7052 | 0.6493 | 0.6372 |
| 28 | 0.6637 | 0.691 | 0.6283 | 0.678 |
| 29 | 0.6777 | 0.6866 | 0.6238 | 0.6395 |
| 30 | 0.7283 | 0.7626 | 0.7035 | 0.7599 |
| 31 | 0.661 | 0.6617 | 0.6961 | 0.6644 |

**Supplementary Table 4.** Correlation coefficients between single-subject maps and the group-average map of the IFG IC cluster for Scan 1 and Scan 2 of the listening-reciting task. For each hemisphere in each subject, a correlation coefficient is computed by the function *Corr* (Single-subject map, Group-average map) in MATLAB. LH: left hemisphere; RH: right hemisphere. Bold numbers indicate low correlation ($r < 0.2$).

| Scan | Scan 1: Subject 1 (Fig. 5A) and Subjects 2-31 (Suppl. Fig. 5) vs. Group-average (Fig. 5B) | | Scan 2: Subject 1 (Fig. 5A) and Subjects 2-31 (Suppl. Fig. 6) vs. Group-average (Fig. 5B) | |
|---|---|---|---|---|
| Subject | LH | RH | LH | RH |
| 1 | 0.5187 | 0.4959 | 0.4814 | 0.4759 |
| 2 | 0.6891 | 0.4069 | 0.7053 | 0.5136 |
| 3 | 0.5036 | 0.3229 | 0.5794 | 0.3911 |
| 4 | 0.5848 | 0.4768 | 0.6224 | 0.4995 |
| 5 | 0.5588 | 0.341 | 0.2228 | **0.0514** |
| 6 | 0.2653 | **0.1717** | 0.2714 | **0.1583** |
| 7 | 0.6096 | 0.4939 | 0.628 | 0.4967 |
| 8 | 0.5469 | 0.3313 | 0.5596 | 0.465 |
| 9 | 0.6698 | 0.4408 | 0.576 | 0.2892 |
| 10 | 0.5292 | 0.3862 | 0.5596 | 0.4677 |
| 11 | 0.5774 | 0.4335 | 0.6305 | 0.4564 |
| 12 | 0.543 | 0.3619 | 0.3591 | 0.2429 |
| 13 | 0.4706 | 0.4205 | 0.4824 | 0.4699 |
| 14 | 0.6832 | 0.3392 | 0.6005 | 0.548 |
| 15 | 0.6053 | 0.4425 | 0.5347 | 0.3184 |
| 16 | 0.6193 | 0.3771 | 0.6288 | 0.4477 |
| 17 | 0.4055 | **0.1355** | 0.559 | 0.313 |
| 18 | 0.6251 | 0.5131 | 0.6001 | 0.4355 |
| 19 | 0.7079 | 0.3555 | 0.612 | 0.3619 |
| 20 | 0.5347 | 0.3719 | 0.5403 | 0.3353 |
| 21 | 0.6736 | 0.4754 | 0.613 | 0.469 |
| 22 | 0.6514 | 0.4604 | 0.5197 | 0.2887 |
| 23 | 0.5969 | 0.5553 | 0.5098 | 0.4519 |
| 24 | 0.4087 | **0.1982** | 0.5965 | 0.4353 |
| 25 | 0.534 | 0.4125 | 0.7163 | 0.5266 |
| 26 | 0.563 | 0.3184 | 0.5869 | 0.4708 |
| 27 | 0.6674 | 0.504 | 0.5698 | 0.534 |
| 28 | 0.4989 | 0.5617 | 0.5484 | 0.5158 |
| 29 | 0.6328 | 0.5052 | 0.6335 | 0.3595 |
| 30 | 0.5076 | 0.3153 | 0.6397 | 0.5182 |
| 31 | 0.5553 | 0.4042 | 0.5893 | 0.3382 |

**Supplementary Table 5.** Correlation coefficients between single-subject maps and the group-average map of the SMC IC cluster for Scan 1 and Scan 2 of the listening-reciting task. For each hemisphere in each subject, a correlation coefficient is computed by the function *Corr* (Single-subject map, Group-average map) in MATLAB. LH: left hemisphere; RH: right hemisphere. Bold numbers indicate low correlation ($r$ < 0.2).

| Scan | Scan 1: Subject 1 (Fig. 6A) and Subjects 2-31 (Suppl. Fig. 7) vs. Group-average (Fig. 6B) | | Scan 2: Subject 1 (Fig. 6A) and Subjects 2-31 (Suppl. Fig. 8) vs. Group-average (Fig. 6B) | |
|---|---|---|---|---|
| Subject | LH | RH | LH | RH |
| 1 | 0.8047 | 0.865 | 0.8219 | 0.867 |
| 2 | 0.6567 | 0.7283 | 0.6881 | 0.7437 |
| 3 | 0.7609 | 0.7992 | 0.7834 | 0.7943 |
| 4 | 0.6432 | 0.6754 | 0.6097 | 0.6314 |
| 5 | 0.7745 | 0.766 | 0.7539 | 0.7583 |
| 6 | 0.6557 | 0.6764 | 0.5094 | 0.5412 |
| 7 | 0.7046 | 0.7373 | 0.7591 | 0.7605 |
| 8 | 0.7805 | 0.7902 | 0.8075 | 0.7991 |
| 9 | 0.6834 | 0.7353 | 0.7087 | 0.7762 |
| 10 | 0.6629 | 0.7106 | 0.7058 | 0.7615 |
| 11 | 0.7799 | 0.7961 | 0.7723 | 0.7773 |
| 12 | 0.6948 | 0.7809 | 0.7366 | 0.8081 |
| 13 | 0.2602 | 0.6126 | **0.0565** | 0.6288 |
| 14 | 0.8413 | 0.8108 | 0.7292 | 0.6977 |
| 15 | 0.6416 | 0.6779 | 0.7255 | 0.7518 |
| 16 | 0.7353 | 0.6785 | 0.713 | 0.6319 |
| 17 | 0.7168 | 0.7632 | 0.7186 | 0.7917 |
| 18 | 0.5844 | 0.5198 | 0.7446 | 0.6947 |
| 19 | 0.6919 | **0.1121** | 0.6457 | **0.017** |
| 20 | 0.7106 | 0.7635 | 0.6901 | 0.7712 |
| 21 | 0.6716 | 0.6093 | 0.8062 | 0.7676 |
| 22 | 0.5086 | 0.4706 | 0.4941 | 0.4188 |
| 23 | 0.7145 | 0.7428 | 0.6998 | 0.7522 |
| 24 | 0.6644 | 0.6672 | 0.6827 | 0.6874 |
| 25 | 0.7771 | 0.7733 | 0.8091 | 0.8019 |
| 26 | 0.6339 | 0.567 | 0.6462 | 0.5764 |
| 27 | 0.6507 | 0.6693 | 0.712 | 0.7448 |
| 28 | 0.6957 | 0.7272 | 0.6445 | 0.6795 |
| 29 | 0.7638 | 0.76 | 0.6661 | 0.6628 |
| 30 | 0.7728 | 0.8218 | 0.7808 | 0.8325 |
| 31 | 0.7576 | 0.6547 | 0.7744 | 0.6468 |
